# Validation of Algorithms Used to Identify Red Blood Cell Transfusion Related Admissions in Veteran Patients with End Stage Renal Disease

**DOI:** 10.5334/egems.257

**Published:** 2019-07-03

**Authors:** Celena B. Peters, Jared L. Hansen, Ahmad Halwani, Monique E. Cho, Jianwei Leng, Tina Huynh, Zachary Burningham, John Caloyeras, Tara Matsuda, Brian C. Sauer

**Affiliations:** 1 Informatics, Decision-Enhancement, and Analytic Sciences Center (IDEAS), VA Salt Lake City Health Care System, Salt Lake City, UT, US; 2 University of Utah, Department of Internal Medicine, Salt Lake City, UT, US; 3 Amgen Inc., US

**Keywords:** Electronic Health Records, Data Collection, Chronic Kidney Failure, Validation Studies, End-Stage Kidney Disease

## Abstract

**Background:**

The goal of this study was to compare the performance of several database algorithms designed to identify red blood cell (RBC) Transfusion Related hospital Admissions (TRAs) in Veterans with end stage renal disease (ESRD).

**Methods:**

Hospitalizations in Veterans with ESRD and evidence of dialysis between 01/01/2008 and 12/31/2013 were screened for TRAs using a clinical algorithm (CA) and four variations of claims-based algorithms (CBA 1–4). Criteria were implemented to exclude patients with non-ESRD-related anemia (e.g., injury, surgery, bleeding, medications known to produce anemia). Diagnostic performance of each algorithm was delineated based on two clinical representations of a TRA: RBC transfusion required to treat ESRD-related anemia on admission regardless of the reason for admission (labeled as TRA) and hospitalization for the primary purpose of treating ESRD-related anemia (labeled TRA-Primary). The performance of all algorithms was determined by comparing each to a reference standard established by medical records review. Population-level estimates of classification agreement statistics were calculated for each algorithm using inverse probability weights and bootstrapping procedures. Due to the low prevalence of TRAs, the geometric mean was considered the primary measure of algorithm performance.

**Results:**

After application of exclusion criteria, the study consisted of 12,388 Veterans with 26,672 admissions. The CA had a geometric mean of 90.8% (95% Confidence Interval: 81.8, 95.6) and 94.7% (95% CI: 80.5, 98.7) for TRA and TRA-Primary, respectively. The geometric mean for the CBAs ranged from 60.3% (95% CI: 53.2, 66.9) to 91.8% (95% CI: 86.9, 95) for TRA, and from 80.7% (95% CI: 72.9, 86.7) to 96.7% (95% CI: 94.1, 98.2) for TRA-Primary. The adjusted proportions of admissions classified as TRAs was 3.2% (95% CI: 2.8, 3.8) and TRA-Primary was 1.3% (95% CI: 1.1, 1.7).

**Conclusions:**

The CA and select CBAs were able to identify TRAs and TRA-primary with high levels of accuracy and can be used to examine anemia management practices in ESRD patients.

## 1. Introduction

In 2011, the Centers for Medicare and Medicaid Services (CMS) changed their reimbursement policy for dialysis centers. Before 2011, certain items, including erythropoiesis-stimulating agents (ESAs) and iron for anemia, were billed separately as fee-for-service. Starting in 2011, CMS bundled ESAs into the case-mix-adjusted bundled payment system [1]. Blood and blood products, however, continue to be billed separately. Studies evaluating the impact of dialysis-related regulatory and reimbursement changes are finding decreases in the rate of ESA utilization, increases in the percentage of patients with Hgb <10 g/dL [2], and an increase in the rate of red blood cell (RBC) transfusions [3,4,5]. Furthermore, some reports indicate a shifting of RBC transfusions from the outpatient to inpatient environments, which may shift costs from dialysis centers to other sites of care [6].

These studies, however, did not adequately isolate the indication for RBC transfusions, making it difficult to estimate the true prevalence of hospital transfusions as an end stage renal disease (ESRD)-related anemia management strategy [4,6,7]. For example, most studies did not exclude RBC transfusions for bleeding, surgery, or inpatient iatrogenic causes. To understand how CMS reimbursement changes impact anemia management practices, we need to accurately measure those admissions that require immediate transfusions to correct ESRD-related anemia. Nevertheless, to our knowledge, no validated *clinical* or *claims-based algorithms* exist to help identify hospital admissions where RBC transfusions were used to treat patients admitted with ESRD-related anemia.

To close this gap, we evaluated two classes of database algorithms to identify Transfusion-Related Admissions (TRAs) in Veterans with dialysis-dependent ESRD. The *clinical algorithms* (CAs), leverage the rich clinical data available within the Veterans Health Administration (VHA) system. The *claims-based algorithms* (CBAs), explore measurement rules typical in administrative databases, such as Medicare and commercial health insurers.

The objective of this study was to determine the performance of the *clinical* and *claims-based algorithms* designed to identify TRAs in Veterans with ESRD and receiving dialysis.

## 2. Methods

We used an agreement design [8] to evaluate the performance of the *clinical* and *claims-based algorithms* against chart-review. We produced the algorithms from historical data generated through routine care. We produced the reference standard, used to determine the performance of the algorithms, through structured chart-review by trained clinical reviewers.

### 2.1. Data and Setting

#### 2.1.1. Data Sources

This study used Veterans Affairs (VA) and Medicare data from 01/01/2008 to 12/31/2013 to evaluate the performance of the clinical and administrative TRA algorithms. The VA Corporate Data Warehouse (CDW) contains data for care provided by the VA (VA care) and outsourced care (VA fee-basis). The CDW contains nearly all clinical and administrative data found in the electronic health record for VA care but is limited to administrative data for VA fee-basis care [9]. Since the electronic health records were used to validate the TRA algorithms, we evaluated their performance in hospitalizations that occurred in the VA care setting. Data from Medicare and VA fee-basis (i.e., care provided outside of the VA but paid by VA) [9] were used to produce a more comprehensive profile of Veteran patients admitted to VA inpatient facilities during the study period. Access to Medicare data is delayed by approximately 24-months before they are available for VA research. Medicare data were only available up to 12/31/2013 when the study began, which limited the study duration. The VA CDW data domains used for inclusion/exclusion criteria and algorithm construction included: procedures, conditions, laboratory and chemistry, vital signs, patient demographics, pharmacy, and orders.

We used the Compensation and Pension Records Interchange (CAPRI) system, which provides National “read only” access to Veteran electronic health records, for chart-review validation [10]. The research was approved by the University of Utah Institutional Review Board (IRB_00076613) and reviewed by the Salt Lake City VA Research Review Committee.

### 2.2. Study Criteria and Measurement

#### 2.2.1. Eligibility Criteria

All VA hospital admissions occurring in Veterans with an ESRD diagnosis and evidence of dialysis in the six months prior to admission met the initial inclusion criteria. Admissions in Veterans with alternative etiologies of anemia (e.g., bleeding, malignancy, surgery, or hematological disorders) were excluded. Healthcare Cost and Utilization Project (HCUP) Single-level Clinical Classification Software (CCS) [11] was used to identify exclusion condition codes and report the discharge principal diagnoses. For a full list of eligibility criteria and coding strategies, please refer to Appendix A in the Supplemental Digital Content.

### 2.3. TRA Identification Algorithms

#### 2.3.1. Clinical Algorithm

The *clinical algorithm* defined a TRA as a hospital admission with evidence of anemia (hemoglobin <9.0 g/dL) 24 hours prior to admission or 24 hours after admission **AND** a RBC transfusion occurring after the hemoglobin measurement **AND** within 24 hours after admission. Measurement details are provided in Section 2 of Appendix A for all algorithms.

#### 2.3.2. Claims-Based Algorithms

We evaluated how well four *claims-based algorithms* identified TRAs in typical administrative claims data. The four *claims-based algorithms* map to the two definitions of TRA described in Section 3.4 below. The first, more inclusive definition represented anemia management with RBC transfusion regardless of the reason for hospitalization (labeled *TRA*). The second represented anemia management with RBC transfusion for Veterans hospitalized primarily for ESRD-related anemia (labeled *TRA-primary*).

1. *Claims-Based Algorithm 1 – Primary ESRD:* Admission with a principal discharge diagnosis of chronic kidney disease (CKD) or ESRD **AND** a secondary diagnosis of anemia **AND** a procedure code for a RBC transfusion during the first two days of admission. This algorithm reflects standard coding practices for anemia in ESRD/CKD.
2. *Claims-Based Algorithm 2 – Any ESRD:* Any discharge diagnosis for CKD or ESRD **AND** any discharge diagnosis for anemia **AND** a procedure code for a RBC transfusion during the first two days of admission.
3. *Claims-Based Algorithm 3 – Primary Anemia:* Admission with a principal discharge diagnosis of anemia **AND** a procedure code for a RBC transfusion during the first two days of admission. This algorithm captures deviation from standard coding practices for patients admitted for anemia management.
4. *Claims-Based Algorithm 4 – Any Anemia:* Admission with any discharge diagnosis for anemia **AND** a procedure code for a RBC transfusion during the first two days of admission.

### 2.4. Development of the Reference Standard

The *reference standard* was established by human chart-review and used to evaluate the performance of the *clinical* and *claims-based algorithms*.

#### 2.4.1. Development of the Electronic Chart-Review Form

The electronic chart-review form guided the reviewers though the chart abstraction steps and captured standardized responses. The form was revised and tested during a series of meetings with clinical experts (MC, AH, CP) and the two chart reviewers (a PharmD with 15+ years of chart-review experience in the VA and 3^rd^ year resident in General Surgery). The form was pre-populated from VA clinical data with patient identifiers, date of admission, and the lowest hemoglobin value during the 24 hours before and after the admission.

Chart-reviewers used the national CAPRI system to view patient charts and answer questions to determine whether a TRA occurred and whether the TRA was the primary reason for the hospital admission. Reviewers focused on the discharge summary, history and physical, and nephrology notes (when available), and were allowed to search other notes as necessary. Chart-abstraction details and images of the user interface are provided in Appendix C.

#### 2.4.2. Sampling of Admissions for Chart-Review

We devised a sampling strategy that supported population-level estimates of algorithm performance while providing insight into where errors may be occurring with the *clinical algorithm* [12].

In addition to the *clinical algorithm* used to define a TRA, we included three variants, two of which were intended to identify potential measurement problems with hemoglobin and RBC transfusions:

1. *Sampling Rule 1:* (*clinical algorithm* for TRA) evidence of anemia (HGB <9.0 g/dL) 24 hours prior to admission or 24 hours after admission **AND** a RBC transfusion occurring after the hemoglobin measurement **AND** within 24 hours after admission.
2. *Sampling Rule 2:* Evidence of anemia 24 hours prior to admission or 24 hours after admission **AND** no evidence of a RBC transfusion within the first 24 hours after admission. This sampling rule identifies potential measurement problems with RBC transfusions.
3. *Sampling Rule 3:* No evidence of anemia 24 hours prior to admission or 24 hours after admission **AND** evidence of a RBC transfusion within the first 24 hours after admission. This sampling rule identifies potential laboratory measurement problems.
4. *Sampling Rule 4:* No evidence of anemia 24 hours prior to admission or 24 hours after admission **AND** no evidence of a RBC transfusion within the first 24 hours of the admission. This sampling rule finds TRA true negatives.

The sample size requirement for chart-review was determined by defining the target level of accuracy as 90% with the lower bound of the 95% confidence interval (CI) being 85%. The minimum sample size requirement was calculated from a standard binomial probability distribution.

When we assumed the point estimate of accuracy or the geometric mean was 90%, a sample size of at least 150 true positive TRAs from *Sampling Rule 1* was required to determine a minimum accuracy of 85% (i.e., the lower bound of the 95% CI was 85%). We intentionally oversampled because we planned at least one formal error analysis after the first batch of 225 admissions. Subsequently, we randomly sampled without replacement; 400 hospitalizations from *Sampling Rule* 1, 200 from each *Sampling Rules* 2 and 3, and 100 from S*ampling Rule* 4. The sample from rule 4 was smaller because we did not expect many false negatives since this rule represents no evidence of anemia AND no evidence of RBC transfusion during the first 24 hours of the hospitalization.

#### 2.4.3. Error Analysis and Rule Revision

The 900 reviewed hospitalizations were initially divided into a set for error analysis (n = 225) and a set for validation (n = 675). The first batch investigated causes for discordance between chart-review and the *sampling rules* to inform revision to the rules and exclusion criteria. Thirty-seven discordant cases between the *sampling rules* and chart-review occurred in the first batch of reviewed admissions. These were classified as either false negative or false positive. Of the four false negatives, two were due to hemoglobin levels meeting anemia criteria not found by the *sampling rules* (*i.e., clinical algorithm*), and two were due to lack of evidence for RBC transfusions when evidence of administration was available in CAPRI. Of the 33 false positives, 15 were attributable to blood loss due to other conditions, seven were due to hematological disorders, and two were due to surgical procedures. Nine false positives were due to transfusions identified in the *sampling rules* but with no evidence of RBC administration from blood bank records. Of these nine false positives, five were detected only via evidence of RBC transfusion orders, while the remaining four were present as procedure codes.

As a result of the error analysis, we added procedure codes for RBC transfusions that were initially missed, reviewed our laboratory mapping procedures, and modified the exclusion criteria to better capture sources of bleeding and surgical procedures. We also extended exclusion criteria to three weeks prior to the hospital admission instead of only including admission procedures and codes as exclusion criteria.

In addition, we evaluated measure performance with and without the inclusion of RBC transfusion orders and found better overall performance when including RBC transfusion orders (sample geometric mean 87.1% vs 86.6%, respectively). The refinement of the exclusion criteria affected our initial validation set of 675 admissions since the additional exclusion criteria resulted in the elimination of 142 unique hospital admissions. The remaining 533 chart-reviewed hospitalizations were used for the formal validation study reported in the results Appendix B.

### 2.5. Statistical Methods

Typical epidemiological measures of classification performance were calculated by comparing each database TRA algorithm against chart-review determination of TRA and TRA-primary, which included accuracy, positive predictive value (PPV), negative predictive value (NPV), specificity (SP), and sensitivity (SE). Since accuracy is heavily dependent upon the population-level proportion of events [13], and a test of a rare event may have high accuracy even if SE is low, the geometric mean of SE and SP was used to measure overall algorithm performance. This measure, the geometric mean, is commonly used in machine learning and balances the importance of SE and SP, allowing the selection of models with superior performance in detecting rare events [12, 14,15,16,17,18].

Inverse probability weighting (IPW) was used to estimate population-level performance metrics. Population-level statistics were calculated by applying sampling weights based on the distribution of the sampling rules in the full study population and then bootstrapping to obtain 95% CIs [19]. The bootstrapped CI occasionally exceeded the upper bound of a binomial probability. To correct for this violation of assumptions, a logit transformation was applied to CIs. The population prevalence of TRA events was estimated based on the common formula (below) that adjusts TRA indicator positives (TRA^+^) with performance characteristics of the database measure, specifically SE and SP [20,21,22]:

{Population\, Prevalence_{adj}} = \left( {{TRA^ + } + SP - 1} \right)/\left( {SE + SP - 1} \right)

The population-level prevalence in this study represents the adjusted proportion of hospital admissions in our study population that were true TRA or TRA-Primary (Appendix A).

## 3. Results

### 3.1 Study Population

Our initial study cohort identified 149,825 Veteran patients with evidence of ESRD between 01/01/2008 and 12/31/2013. We identified 98,209 hospital admissions in 24,856 Veterans who met initial inclusion criteria for ESRD and had evidence of dialysis within 6 months prior to the hospital admission (Table 1). The final study population included 26,672 admissions from 12,388 Veteran patients.

**Table 1:** Attrition table describing population attrition based on exclusion criteria.

Characteristics	Patient	Hospitalizations

Eligibility: ESRD Diagnosis	149,825	NA
Eligibility: ESRD Diagnosis and Dialysis	78,467	NA
Inclusion: Hospital Admissions	31,515	132,357
Inclusion: Hospital Admissions with prior ESRD	27,328	112,265
Inclusion: Hospital Admissions with prior ESRD and Dialysis	24,856	98,209
Exclusion: Malignancy Rx	24,770	97,520
Exclusion: Admission Hematological Dx	23,996	93,741
Exclusion: Admission GI Bleed	19,804	77,237
Exclusion: Admission Injury	15,254	42,880
Exclusion: Station = Las Vegas	15,220	42,712
Exclusion: Admission Major Surgery	14,441	36,221
Exclusion: Admission Malignancy Dx	13,647	30,606
Exclusion (Amended): Lupus (3-weeks)	13,579	30,427
Exclusion (Amended): Any Surgery	13,312	29,670
Exclusion (Amended): Hematological (3-weeks)	13,026	28,743
Exclusion (Amended): GI bleed (3-weeks)	12,388	26,672
Total Study Population	12,388	26,672

*Note*: The number of patients and admissions remaining after each exclusion are presented in the above table.

### 3.2. Study Population Characteristics

Our 12,388 Veterans with a hospital admission during the study period were predominately male 98.1% (95% CI: 97.8%, 98.3%) with an average age of 66.3 (95% CI: 66.1, 66.5); and the majority had diabetes [77% (95% CI: 76.2%, 77.7%)], hypertension [99.2% (95% CI: 90%, 99.3%)], dyslipidemia [75.5% (95% CI: 74.7%, 76.2%)], heart failure [62.4% (95% CI: 61.5%, 63.2%)] and coronary artery disease [72.4% (95% CI: 71.6%, 73.2%)] (Table 2).

**Table 2:** Basic description of the study population.

	Count	Mean Percent	95% CI

Age	12,388	66.3	(66.1, 66.5)
Male	12,148	98.1%	(97.8%, 98.3%)
Female	238	1.9%	(1.7%, 2.2%)
Unknown Sex	2	0.0%	(0.0%, 0.1%)
White	6,453	52.1%	(51.2%, 53.0%)
Black	4,993	40.3%	(39.4%, 41.2%)
Other Races	428	3.5%	(3.1%, 3.8%)
Unknown Race	514	4.1%	(3.8%, 4.5%)
Diabetes Mellitus	9,534	77.0%	(76.2%,77.7%)
Hypertension	12,285	99.2%	(99.0%, 99.3%)
Dyslipidemia	9,347	75.5%	(74.7%, 76.2%)
Heart Failure	7,727	62.4%	(61.5%, 63.2%)
Coronary Artery Disease	8,972	72.4%	(71.6%, 73.2%)
Peripheral Artery Disease	4,878	39.4%	(38.5%, 40.2%)
Atrial Fibrillation	3,551	28.7%	(27.9%, 29.5%)
Arrhythmia	6,036	48.7%	(47.8%, 49.6%)
Stroke	3,776	30.5%	(29.7%, 31.3%)

95% CI = 95% Confidence Interval.

### 3.3. Sampling Strategy and Weights for Chart-Review

The four sampling rules were applied to the 26,672 hospital admissions that met full study inclusion criteria. After changes to the exclusion criteria were applied, 533 of the initial 675 charts reviewed remained eligible for evaluation. We did not anticipate major changes to the exclusion criteria and resulting study population and therefore did not conduct chart-review in phases that would have allowed us to change our sampling strategy as a result of changes in the eligible study population. See Table 3.

**Table 3:** Sampling strategy for chart-review and weights.

Sampling Rule	N Adm.	N Sampled	P (Sampled)	Weight

1: Anemia & RBC-T	993	237	0.239	4.19
2: Anemia & No RBC-T	3531	122	0.035	28.94
3: No anemia & RBC-T	165	111	0.673	1.49
4: No anemia & RBC-T	21983	63	0.003	348.94

RBC-T = Red Blood Cell Transfusion. N Adm. = Number of hospital admissions in study population by sampling rule. N Sampled = Number randomly sampled from each sampling rule for chart-review. P(Sampled) = Probability of being sampled from admissions that screened positive for each sampling rule.

**Figure 1:**
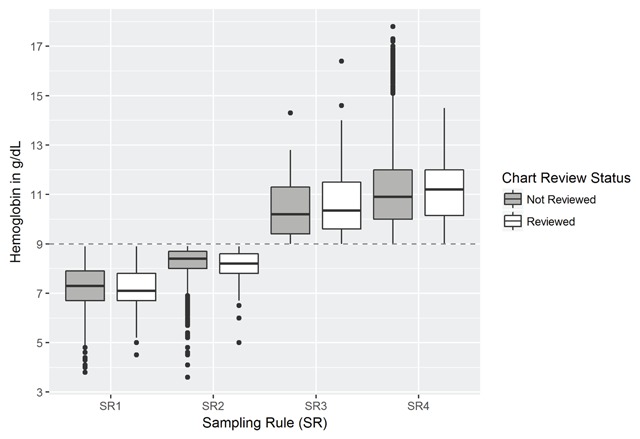
Hemoglobin levels by sampling rule and chart-review status.

The average hemoglobin for *Sampling Rule 1*(clinical algorithm for TRA) was 7.3 g/dL (95% CI:7.2, 7.3) for admissions not reviewed and 7.2 g/dL (95% CI: 7.1, 7.3) for the admissions randomized to chart-review. *Sampling Rule 2* (evidence of anemia but not RBC transfusion) had a slightly higher average hemoglobin 8.3 g/dL (95% CI: 8.2, 8.3) and 8.1 g/dL (95% CI: 8.0, 8.2) in both the non-reviewed and the chart-review groups, respectively. The average hemoglobin levels for *Sampling Rules 3–4*, which did not meet criteria for anemia, were above 10 g/dL for both chart-reviewed and the other hospital admissions (Figure 1). Hemoglobin measures were missing in six admissions from *Sampling Rule 3* and 1,593 admissions from *Sampling Rule 4*.

### 3.4. Population-Level Performance of Database Algorithms for TRA

Table 4 provides estimates of the population-based performance metrics for the *clinical algorithm* (CA) and the four *claims-based algorithms* (CBAs) based on the more inclusive *TRA* definition. Recall that, for this definition of TRA, the chart-reviewers were instructed to assume the anemia was related to ESRD unless alternative evidence was documented in the notes (e.g., injury causing bleeding) and the RBC transfusion was the assumed management strategy for ESRD related anemia unless other evidence was observed in the notes (e.g., a statement that the RBC transfusion was used prophylactically prior to surgery and the reviewer saw no evidence that the treating provider would have treated the apparent anemia).

**Table 4:** Population-level algorithm performance and estimated prevalence for TRA reported as percentages.

	CA(TRA)	CBA 1 (TRA)	CBA 2 (TRA)	CBA 3(TRA)	CBA 4 (TRA)

Positive predictive value [% (95% CI)]	72.1 (66–77.4)	80 (63.9–90.1)	66.1 (56.4–74.5)	78.9 (70–85.7)	65.7 (56.7–73.8)
Negative predictive value	99.4 (98.7–99.8)	98 (97.6–98.3)	99.3 (99–99.6)	97.9 (97.3–98.4)	99.5 (99.2–99.7)
Sensitivity	83.5 (67.9–92.4)	38.3 (29.3–48.3)	80.6 (71.7–87.2)	36.6 (28.7–45.2)	85.5 (76.6–91.5)
Specificity	98.9 (98.7–99.1)	99.7 (99.3–99.8)	98.6 (98–99)	99.7 (99.5–99.8)	98.5 (97.9–98.9)
Accuracy	98.4 (97.8–98.9)	97.7 (97.2–98.1)	98 (97.4–98.5)	97.6 (97–98.1)	98.1 (97.5–98.5)
Geometric mean	90.8 (81.8–95.6)	61.7 (53.7–69.1)	89.1 (84–92.8)	60.3 (53.2–66.9)	91.8 (86.9–95)
Unadjusted prevalence	3.7 (3.5–4.0)	1.3 (1.2–1.4)	3.6 (3.4–3.8)	1.5 (1.4–1.7)	3.8 (3.6–4.1)
Adjusted prevalence	3.2 (2.8–3.8)	3.2 (2.8–3.8)	3.2 (2.8–3.8)	3.2 (2.8–3.8)	3.2 (2.8–3.8)

CA: Clinical Algorithm, CBA1–4: Claims-based Algorithm 1–4, TRA: Transfusion Related Admission (inclusive).

The geometric mean was the primary measure of performance for the database algorithms designed to identify ESRD-related TRAs. The geometric mean for TRAs ranged from 60.3% (95% CI: 53.2, 66.9) for CBA 3 to 91.8% (95% CI: 86.9, 95) for CBA 4. The CA had a geometric mean of 90.8% (95% CI: 81.8, 95.6) and accuracy of 98.4% (95% CI: 97.8, 98.9). The CBAs requiring principal discharge diagnoses of CKD/ESRD (CBA 1) and anemia (CBA 3) had the lowest geometric means 61.7% (95% CI: 53.7, 69.1) and 60.3% (95% CI: 53.2, 66.9), respectively. They also had the greatest discrepancy between geometric means and accuracy. Table 4 provides additional population-level measures of algorithm performance required to compute population-level TRA prevalence, which was estimated to be 3.2% (95% CI: 2.8, 3.8) during the study period.

### 3.5. Population-Level Performance of Database Algorithms for TRA-Primary

Table 5 provides population-based performance metrics for the CA and the four CBAs based on the more restrictive TRA-Primary definition. Recall that, for TRA-Primary, chart-reviewers were asked whether the ESRD-related anemia was the primary reason for the hospital admission. The geometric mean for TRA-Primary ranged from 80.7% (95% CI: 72.9, 86.7) for CBA 1 to 96.7% (95% CI: 94.1, 98.2) for CBA 4. The CA had a geometric mean of 94.7% (95% CI: 80.5, 98.7) and accuracy of 97.4% (95% CI: 97.1, 97.7). The CBAs requiring principal discharge diagnoses of CKD/ESRD (CBA 1) and anemia (CBA 3) had the lowest geometric means 80.7% (95% CI: 72.9, 86.7) and 88.6% (95% CI: 77.7, 94.6), respectively. They also had the greatest discrepancy between accuracy and geometric means, but were more similar than these discrepancies for the more inclusive TRA (Table 5).

**Table 5:** Population-level algorithm performance and estimated prevalence for TRA-Primary, reported as percentages. Note that unadjusted prevalence is the same for Tables 4 and 5, as it is based solely on screening positive using a given algorithm.

	CA (TRA-Primary)	CBA 1 (TRA-Primary)	CBA 2 (TRA-Primary)	CBA 3 (TRA-Primary)	CBA 4 (TRA-Primary)

Positive predictive value [% (95% CI)]	32.8 (27.1–39.1)	57.2 (40.4–72.5)	31.5 (24.3–39.8)	70.5 (60.9–78.6)	30.7 (23.8–38.5)
Negative predictive value	99.9 (99.2–100)	99.5 (99.4–99.7)	99.9 (99.8–100)	99.7 (99.3–99.9)	100 (99.9–100)
Sensitivity	92.1 (62.6–98.8)	65.8 (53.9–75.9)	92.9 (85.1–96.8)	79.1 (61.4–90)	96.5 (89.7–98.8)
Specificity	97.5 (97.2–97.7)	99.3 (98.8–99.6)	97.3 (96.6–97.8)	99.6 (99.4–99.7)	97 (96.4–97.6)
Accuracy	97.4 (97.1–97.7)	98.9 (98.4–99.2)	97.2 (96.5–97.7)	99.3 (98.9–99.5)	97 (96.4–97.6)
Geometric mean	94.7 (80.5–98.7)	80.7 (72.9–86.7)	95 (91.3–97.2)	88.6 (77.7–94.6)	96.7 (94.1–98.2)
Unadjusted Prevalence	3.7 (3.5–4.0)	1.3 (1.2–1.4)	3.6 (3.4–3.8)	1.5 (1.4–1.7)	3.8 (3.6–4.1)
Adjusted Prevalence	1.3 (1.1–1.7)	1.3 (1.1–1.7)	1.3 (1.1–1.7)	1.3 (1.1–1.7)	1.3 (1.1–1.7)

CA: Clinical Algorithm, CBA1–4: Claims-based Algorithm 1–4, TRA-Primary: Transfusion Related Admission where ESRD anemia thought to be the cause of the hospital admission.

### 3.6. Principal Discharge Diagnoses by TRA Type from Chart-Reviewed Admissions

The discharge summary principal diagnoses differed substantially by TRA designation. Admissions identified as TRA-Primary grouped into four principal discharge diagnoses based on HCUP CCS categories: *deficiency and other anemia* (73), *hypertension* with complications and *secondary hypertension* (2), *CKD* (3), and *syncope* (1). The more inclusive definition of TRAs grouped into 32 CCS categories, with *deficiency and other anemia* (82), *hypertension with complications and secondary hypertension* (13), and *congestive heart failure* (10) representing the top three reasons for the hospital admission. Admissions classified as not being TRAs grouped into 94 different CCS categories. Table 6 lists the most frequent CCS categories by TRA-primary and TRA.

**Table 6:** Abbreviated list of principal diagnosis codes from discharge summary by TRA type (TRA, TRA-primary, and No TRA).

CCS No.	CCS Label	TRA-Primary	TRA	No TRA

59	Deficiency and other anemia	73	82	30
99	Hypertension with complications and secondary hypertension	2	13	30
108	Congestive heart failure; non-hypertensive	0	10	23
2	Septicemia (except in labor)	0	8	12
55	Fluid and electrolyte disorders	0	8	20
158	Chronic kidney disease	3	7	39
100	Acute myocardial infarction	0	4	16
106	Cardiac dysrhythmias	0	4	9
50	Diabetes mellitus with complications	0	3	9
199	Chronic ulcer of skin	0	3	2
157	Acute and unspecified renal failure	0	3	6
259	Residual codes; unclassified	0	3	5
660	Alcohol-related disorders	0	2	1
254	Rehabilitation care; fitting of prostheses; and adjustment of devices	0	2	10
248	Gangrene	0	2	3
159	Urinary tract infections	0	2	4
245	Syncope	1	1	3

CCS: HCUP Clinical Classification System.

## 4. Discussion

This study compared the classification performance of multiple database algorithms designed to identify *transfusion related admissions* (TRAs) within the Veteran population, using two categorization criteria aimed at identifying ESRD-specific events from other reasons to transfuse. Recently, a series of related studies have provided notable real-world evidence of how top-down CMS policy and reimbursement changes impact ESRD-related anemia management practices. Interpreting the effect of these changes on the use of RBC transfusions for anemia management, however, is difficult due to lack of validated criteria for attributing RBC transfusions to ESRD-related anemia. The previous studies often lacked exclusion criteria for alternative explanations of anemia (e.g., gastrointestinal (GI) bleeding and surgery) and did not use laboratory data or medical codes to indicate anemia was present on admission. Furthermore, they made no attempt to establish rules linking transfusion procedures to hospital admission dates to differentiate community onset vs. nosocomial anemia – an important distinction since CMS reimbursement changes may incentivize cost shifting from dialysis facilities to hospital-based environments [6,23]. Our study addressed these measurement limitations so future studies can more accurately evaluate the impact of changes in anemia management policies and practices. Our *clinical algorithm* (CA) performed well for both TRA concepts, with a geometric means above 90% for TRA and TRA-Primary. The CBAs that did not require principal diagnoses of CKD/ESRD (CBA 2) or anemia (CBA 4) on the discharge diagnoses performed better than the CBAs that required principal discharge diagnoses of CKD/ESRD (CBA 1) and anemia (CBA 3).

Surprisingly, the CBA 2 and CBA 4 performed as well or slightly better than the CA, indicating that clinical laboratory data may not be needed for identification. Conversely, the CA used laboratory data to identify anemia on admission, and multiple sources of information, such as orders, laboratory data, and procedure codes, were used to identify the administration of RBC transfusions. Error analysis of the validation data indicated that many false positives were due to our lack of ability to adequately exclude patients with alternative explanations of anemia. This was, in part, due to the lack of medical codes supportive of the medical notes, which may have included references to conditions like a GI bleed. Problems with sensitivity, using the CBAs was more often a result of TRAs not including CKD/ESRD or anemia codes rather than missed transfusions.

The CA and all CBAs had excellent specificity for both TRA and TRA-Primary and overall performance was better across all algorithms for TRA-primary compared to the TRA definition. This finding can be intuitively explained because TRA-Primary was more likely to have discharge diagnoses codes consistent with ESRD-related anemia and therefore had a lower proportion of false positives due to alternative reasons for anemia.

The two concepts of TRA defined in our study provide an instrument to investigate whether dialysis facilities are referring patients to hospitals for RBC transfusions to manage anemia; or if patients maintained at lower hemoglobin levels meet clinical requirements for transfusion when presenting to the hospital for acute exacerbations of conditions other than ESRD. Increases in the prevalence of the TRA-Primary may indicate the former, while increases in the prevalence of the more inclusive concept of TRA may indicate the latter.

The hemoglobin distribution among the randomized chart-reviewed admissions and the non-reviewed population in each of the four sampling rules were remarkably similar. The similarities support our sample findings’ utility for estimating population performance and adjusted prevalence using the sampling weights technique employed in this study. Clinical guidelines do not suggest transfusing patients at a hemoglobin threshold and recommend avoiding when possible [24]. In contrast, it is generally recommended that patients with CKD receiving ESAs should not be maintained at hemoglobin levels above 11.5 g/dL and levels above 13 g/dL should never be an intentional target [24]. However, studies investigating the reasons transfusions are ordered report low hemoglobin levels as a leading factor. These transfusions typically occurred at values below 11 g/dL with means hovering slightly over 7 g/dL, when low hemoglobin was a factor in the decision making process [25]. Our results are consistent with this work as the mean hemoglobin levels for SR1 reviewed and non-reviewed records (Figure 1) were 7.2 g/dL and 7.3 g/dL.

### 4.1. Contribution to the literature

Any increase in the use of RBC transfusions to treat ESRD-related anemia is alarming since the chronic dialysis population is associated with increased risk of transfusion-related complications, such as hemolytic and non-hemolytic transfusion reactions, infections, and reduction in transplant potential [7]. The previous approaches used to infer increased use of RBC transfusions as a result of CMS reimbursement are limited in their ability to measure important changes in transfusion rates as a result of ESRD-related anemia policy and decisions. When measurement error is unaccounted for, it is more difficult to evaluate the effects of anemia management policy and it is not possible to estimate the true prevalence of TRA and TRA-Primary based on adjustment for measurement performance. In addition, when measurement performance is not incorporated into the analysis, a much larger true effect is needed to identify significant changes in transfusion rates – due to measurement error. The result is also likely an overestimate of the use of RBC transfusions to treat ESRD-related anemia due to alternative causes of bleeding and nosocomial anemia.

As a result of the sampling strategy employed in this study, we were able to investigate key metrics of algorithm performance making it possible to estimate adjusted population prevalence of *TRAs* and *TRA-primary*. This will allow us to more effectively analyze changes in anemia management practices in VA and will inform measurement and validation strategies for application to CMS and other data environments. More broadly, the separation of ESRD specific (*TRA-primary*) and ESRD non-specific (*TRA*) events may complement recently available CMS anemia management measures, such as the Standardized Transfusion Ratio (STrR) [26,27]. The STrR is the ratio of facility level observed RBC transfusions in dialyzed patients to the predicted number that is expected to occur for patients within a facility, which is a crude measure intended to identify facilities that are using RBC transfusions in disproportionate amounts as part of their anemia management strategy.

### 4.2. Limitations

VA is not directly linked to CMS reimbursement criteria, and so may exhibit different coding practices performed by outside facilities. Nevertheless, VA policy requires that all administrative data are accurate and consistent. Administrative coding in VA, however, is primarily used for resource and personnel management along with managing quality and safety of health care delivery. For this reason, the CBAs that heavily rely on discharge diagnoses may not perform as well in CMS data. However, if facilities and providers are following CMS coding guidelines, then we would expect to see similar algorithm performance for TRA-Primary since the algorithms were derived from CMS coding guidelines for ESRD-related anemia management. Performance of the algorithms to measure the more inclusive TRA concept may not be as robust when anemia management is not the primary reason for admission.

Medicare now requires reporting of hemoglobin/hematocrit levels along with dates of service for all ESRD dialysis patient to support the new Quality Incentive Program, rather than only reporting hemoglobin/hematocrit levels for patients administered ESAs, will make it possible to monitor the more inclusive TRAs concept by applying the *clinical algorithm*, in CMS and United States Renal Data Systems (USRDS) data [26]. However, since hospital facilities are not required to submit these laboratory measures, modifications to the *clinical algorithm* may be warranted to account for evidence of anemia reported by dialysis facilities when admissions were not within 24 hours of a dialysis events.

Our initial error analysis found that the majority of false positives were due to missed exclusion criteria, specifically regarding GI bleeding. We often found evidence in the medical note of GI hemorrhage, but patients admitted for another condition may not have had the condition coded in the discharge summary. For this reason, we expanded the exclusion criteria to include clinical data that may not be routinely available in CMS claims data. For example, we included evidence of intravenous (IV) administration of proton pump inhibitors and positive guaiac tests. The algorithm performance estimates are dependent on the population studied and this must be taken into consideration if attempts are made to implement these algorithms in claims-based data marts.

Since VistA Blood Establishment Computer Software (VBECS) data available in CAPRI had a date and not a time stamp, the reference standard was established by allowing the RBC transfusion to occur on the day of admission or the following day. The CBAs also lacked a time stamp since we tried to mimic data we have available from CMS. The CA, on the other hand, used a date-time stamp and required RBC transfusions to occur within 24-hours of admission. This difference may account for the slightly better performance of the two of the CBAs. Additional work is needed to define the optimal time limit for attributing RBC transfusion as treatment for “community-onset” ESRD-related anemia.

As seen in Table 6, nine records with a CCS principal discharge diagnosis were not classified as a TRA-Primary. This runs counter to the definition of a TRA-primary, which is defined as an admission for the primary purpose of ESRD-anemia. To determine the cause of the misalignment between TRA-Primary definition and ICD-9 coding, we performed a post-hoc evaluation and revisited medical notes related to each of the nine discordant cases. We determined that this lack of overlap was likely due to a coding error for eight cases, as the assigned principal diagnosis code (ICD-9) of anemia was not consistent with the stated cause of admission on the discharge summary. An example of this occurred when the patient was admitted for chest pain and found to have chronic obstructive pulmonary disease (COPD). While the anemia was treated during the admission, the COPD was the primary cause of the admission, as detailed in the text, however it was not coded as the principal diagnosis. The variability in the structure of the medical note may have played a role in the miscoding, as there was no standard format across all notes revisited in the post-hoc analysis.

Finally, inclusion/exclusion criteria and CBAs were based on ICD-9 codes. The current coding standard has migrated to ICD-10 codes, which may affect performance of both the CA and CBAs if coding practices do not represent diagnostic intentions. Nevertheless, we anticipated this issue and used HCUP CCS ICD grouping software, which provides a well-documented and maintained crosswalk between ICD-9 and ICD-10. Meaning, the algorithms do not require modification to implement using ICD-10 codes, but additional validation would be prudent to ensure stability of performance.

## 5. Conclusions

Both our CA and select CBAs perform well and can be used to study CMS policy and reimbursement changes. This work also represents a crucial step in generating more knowledge surrounding transfusions related ESRD-anemia in the absence of alternative indications. The measurement of SE and SP supports computation of adjusted prevalence estimates to more accurately reflect the true proportion of admissions meeting TRA and TRA-Primary criteria. We expect our CBAs are portable to other claims-based data sources, nevertheless, validation in new populations is suggested to accurately account for measurement performance to produce adjusted prevalence estimates specific to population being studied.

## Key Points

***We would like to highlight key take-home messages of our work.***

- New policies established by the FDA and CMS may have affected anemia management strategies causing a shift in RBC transfusions, ESAs, and intravenous iron after 2011.
- Related studies do not separate the cause of RBC transfusions is an important step in understanding the true prevalence and implications of these events in ESRD patients with anemia.
- Related studies do not separate the cause of RBC transfusions in ESRD patients; and so, validated methods are needed to estimate the true prevalence of ESRD-related anemia.

## Additional Files

The additional files for this article can be found as follows:

10.5334/egems.257.s1 Appendix A. Database measures.

10.5334/egems.257.s2 Appendix B. Error analysis performed on algorithms prior to study implementation.

10.5334/egems.257.s3 Appendix C. Chart review procedures.
