# Supplementary material for: Validation of Algorithms Used to Identify Red Blood Cell Transfusion Related Admissions in Veteran Patients with End Stage Renal Disease
Source: EGEMS (Wash DC). 2019 Jul 3;7(1):23. doi: 10.5334/egems.257 (PMC6611485; doi:10.5334/egems.257)
Supplement: Appendix A. — Database measures. [file egems-7-1-257-s1.pdf]

Appendix A. Study Criteria Used to Identify and Validate TRA Algorithms

Purpose: The Supplemental Digital Content details all parameters for the Veterans Health Administrations (VHA) transfusion related admissions (TRA) validation study, including data definitions used to define the clinical and claims-based TRA classification algorithms. It is important to note, that determination of search strategy components, (e.g., defining criteria for dialysis or RBC transfusion) underwent a continual process of conducting patient record reviews to determine the potential validity of specific elements of the algorithms. We used the Compensation and Pension Records Interchange (CAPRI) to review patients (in a non-formal way) to confirm how components of the algorithm were working prior to formal validation of each algorithm. An overview of the eligibility criteria is provided. Information is organized by clinical concept and includes rationale, tables, time frames, and vocabulary.
Organization:

1) Section 1: Eligibility Criteria
    a. Clinical Concept: ESRD
        i. Table 1. ESRD: ICD9 Diagnosis Codes
    b. Clinical Concept: Dialysis
        i. Table 2. Dialysis: ICD9 Procedure Codes
        ii. Table 3. Dialysis: CPT/HCPCS Procedure Codes
        iii. Table 4. Dialysis: Stop Codes
    c. Clinical Concept: Exclusion Conditions
        i. Table 5. Exclusion Criteria: ICD9 Diagnosis Codes
        ii. Table 6. Exclusion Criteria: HCUP CCS Codes
        iii. Table 7. Exclusion Criteria: VA Drug Class Codes (Antineoplastics)
    d. Clinical Concept: Anemia
        i. Table 8. Clinical Anemia: LOINC codes used to identify Hemoglobin (HGB) and Hematocrit (HCT)
    e. Clinical Concept: RBC Transfusion
        i. Table 9. Clinical RBC Transfusion: ICD9 Procedure codes used to identify RBC transfusions
        ii. Table 10. Clinical RBC Transfusion: CPT/HCPCS Procedure codes used to identify RBC transfusions
        iii. Table 11. Clinical RBC Transfusion: Orderable Items Used as Evidence of RBC Transfusion
        iv. Table 12. Clinical RBC Transfusion: MCA Feeder Keys Used as Evidence of RBC Transfusion
2) Section 2: Clinical Algorithm TRA
3) Section 3: Claims Based TRA
    a. Table 13. Claims Based Algorithm: ICD9 Inpatient Discharge Diagnoses of CKD, ESRD, or Anemia in CKD
    b. Table 14. ICD9 Inpatient Discharge Diagnoses of Anemia
    c. Table 15. TRA and TRA-Primary concept definitions and four corresponding CBAs designed to identify them in the data
4) Section 4: Sampling Procedures
5) Section 5: Statistical Estimation of Population-Level Parameters
6) Section 6: Document Abbreviation Legend
7) Section 7: Appendix A References

Section 1. Eligibility Criteria

Rationale: The study population is comprised of Veteran patients with end stage renal disease (ESRD) on dialysis with evidence of VHA care within the past 365 days. Patients were eligible for entry into in the study at the first date at which they showed evidence of both ESRD and dialysis during the study period (2007 until 2013, inclusive). This is considered the initial eligibility date used to pull data for our population out of the Corporate Data Warehouse (CDW). Two stages of inclusion criteria were implemented. The first was to extract patients from the CDW and the second was to determine hospital admissions that met inclusion criteria for our study. The unit of analysis was the hospital admission for patients with evidence of ESRD and dialysis within six months of their hospitalization. Additional exclusion criteria were applied in relation to the hospitalization to rule out alternative explanations for anemia

A. Clinical Concept: ESRD

Rationale: Patients with ESRD generally suffer from disease related anemia, which is subject to treatment by ESAs or alternatively RBC transfusions. For this reason, the study defined patients as eligible for inclusion if they had evidence of ESRD and use of dialysis.
Tables:

1. CDW Inpatient Discharge Diagnosis
2. CDW Outpatient Diagnosis
3. Fee-basis Service Provided Diagnosis
4. Fee-basis Inpatient Invoice Diagnosis
5. CMS Inpatient Diagnosis
6. CMS Outpatient Diagnosis

Timeframe:

1. Study Period (to determine patient eligibility)
2. 6 months prior to admission (to determine hospitalization inclusion criteria)

Vocabulary: ICD9 procedure codes

Table 1. ESRD: ICD9 Diagnosis Codes

ICD9 DESCRIPTION	ICD9 CODE
HYPERTENSIVE CHRONIC KIDNEY DISEASE, MALIGNANT, WITH CHRONIC KIDNEY DISEASE STAGE V OR END STAGE RENAL DISEASE	403.01
HYPERTENSIVE CHRONIC KIDNEY DISEASE, BENIGN, WITH CHRONIC KIDNEY DISEASE STAGE V OR END STAGE RENAL DISEASE	403.11
HYPERTENSIVE CHRONIC KIDNEY DISEASE, UNSPECIFIED, WITH CHRONIC KIDNEY DISEASE STAGE V OR END STAGE RENAL DISEASE	403.91
HYPERTENSIVE HEART AND CHRONIC KIDNEY DISEASE, MALIGNANT, WITHOUT HEART FAILURE AND WITH CHRONIC KIDNEY DISEASE STAGE V OR END STAGE RENAL DISEASE	404.02
HYPERTENSIVE HEART AND CHRONIC KIDNEY DISEASE, MALIGNANT, WITH HEART FAILURE AND WITH CHRONIC KIDNEY DISEASE STAGE V OR END STAGE RENAL DISEASE	404.03
HYPERTENSIVE HEART AND CHRONIC KIDNEY DISEASE, BENIGN, WITHOUT HEART FAILURE AND WITH CHRONIC KIDNEY DISEASE STAGE V OR END STAGE RENAL DISEASE	404.12
HYPERTENSIVE HEART AND CHRONIC KIDNEY DISEASE, BENIGN, WITH HEART FAILURE AND CHRONIC KIDNEY DISEASE STAGE V OR END STAGE RENAL DISEASE	404.13
HYPERTENSIVE HEART AND CHRONIC KIDNEY DISEASE, UNSPECIFIED, WITHOUT HEART FAILURE AND WITH CHRONIC KIDNEY DISEASE STAGE V OR END STAGE RENAL DISEASE	404.92
HYPERTENSIVE HEART AND CHRONIC KIDNEY DISEASE, UNSPECIFIED, WITH HEART FAILURE AND CHRONIC KIDNEY DISEASE STAGE V OR END STAGE RENAL DISEASE	404.93
END STAGE RENAL DISEASE	585.6

ESRD = End Stage Renal Disease, ICD9=International Classification of Diseases 9

B. Clinical Concept: Dialysis

Rationale: A record of dialysis was used as part of the eligibility criteria as a marker to confirm the diagnosis of ESRD and to ensure patients were receiving dialysis treatment in the VA.
Tables:
1. CDW Inpatient CPT Procedures
2. CDW Inpatient ICD9 Procedures
3. CDW Inpatient Surgical ICD9 Procedures
4. CDW Outpatient CPT Procedures
5. CDW Outpatient Visit Stop Codes
6. Fee-basis Service Provided CPT Procedures
7. Fee-basis Inpatient Invoice ICD9 Procedures
8. CMS Inpatient ICD9 Procedures
9. CMS Outpatient TOB codes

Timeframe:
1. Study Period (to determine patient eligibility)
2. 6 months prior to admission (to determine hospitalization inclusion criteria)

Vocabulary: ICD9 procedure codes, HCPCS/CPT procedure codes, VHA dialysis clinic visit stop codes 602-611 (except 605)
Additional: Medicare dialysis clinic Type of Billing (TOB) code 72

Table 2. Dialysis: ICD9 Procedure Codes

ICD9 PROCEDURE DESCRIPTION	ICD9 PROCEDURE CODE
HEMODIALYSIS	39.95
HEMODIALYSIS FOR CHRONIC RENAL FAILURE	39.951
HEMODIALYSIS FOR OTHER CONDITIONS	39.953
PERITONEAL DIALYSIS	54.98
PERITONEAL DIALYSIS FOR CHRONIC RENAL FAILURE	54.981
PERITONEAL DIALYSIS FOR OTHER CONDITIONS	54.983

ICD=International Classification of Diseases

Table 3. Dialysis: CPT/HCPCS Procedure Codes

CPT DESCRIPTION	CPT CODE
HEMODIALYSIS PROCEDURE WITH SINGLE PHYSICIAN EVALUATION	90935
HEMODIALYSIS PROCEDURE REQUIRING REPEATED EVALUATION(S) WITH OR WITHOUT SUBSTANTIAL REVISION OF DIALYSIS PRESCRIPTION	90937
DIALYSIS PROCEDURE OTHER THAN HEMODIALYSIS (EG, PERITONEAL DIALYSIS, HEMOFILTRATION, OR OTHER CONTINUOUS RENAL REPLACEMENT THERAPIES), WITH SINGLE PHYSICIAN EVALUATION	90945
DIALYSIS PROCEDURE OTHER THAN HEMODIALYSIS (EG, PERITONEAL DIALYSIS, HEMOFILTRATION, OR OTHER CONTINUOUS RENAL REPLACEMENT THERAPIES) REQUIRING REPEATED PHYSICIAN EVALUATIONS, WITH OR WITHOUT SUBSTANTIAL REVISION OF DIALYSIS PRESCRIPTION	90947
END-STAGE RENAL DISEASE (ESRD) RELATED SERVICES FOR HOME DIALYSIS PER FULL MONTH, FOR PATIENTS YOUNGER THAN 2 YEARS OF AGE TO INCLUDE MONITORING FOR THE ADEQUACY OF NUTRITION, ASSESSMENT OF GROWTH AND DEVELOPMENT, AND COUNSELING OF PARENTS	90963
END-STAGE RENAL DISEASE (ESRD) RELATED SERVICES FOR HOME DIALYSIS PER FULL MONTH, FOR PATIENTS 2-11 YEARS OF AGE TO INCLUDE MONITORING FOR THE ADEQUACY OF NUTRITION, ASSESSMENT OF GROWTH AND DEVELOPMENT, AND COUNSELING OF PARENTS	90964
END-STAGE RENAL DISEASE (ESRD) RELATED SERVICES FOR HOME DIALYSIS PER FULL MONTH, FOR PATIENTS 12-19 YEARS OF AGE TO INCLUDE MONITORING FOR THE ADEQUACY OF NUTRITION, ASSESSMENT OF GROWTH AND DEVELOPMENT, AND COUNSELING OF PARENTS	90965
END-STAGE RENAL DISEASE (ESRD) RELATED SERVICES FOR HOME DIALYSIS PER FULL MONTH, FOR PATIENTS 20 YEARS OF AGE AND OLDER	90966
DIALYSIS TRAINING, PATIENT, INCLUDING HELPER WHERE APPLICABLE, ANY MODE, COMPLETED COURSE	90989
HEMODIALYSIS TRAINING AND/OR COUNSELING	90990
HOME HEMODIALYSIS CARE, OUTPATIENT, FOR THOSE SERVICES EITHER PROVIDED BY THE PHYSICIAN PRIMARILY RESPONSIBLE FOR TOTAL HEMODIALYSIS CARE OR UNDER HIS DIRECT SUPERVISION, AND EXCLUDES CARE FOR COMPLICATING ILLNESSES UNRELATED TO HEMODIALYSIS, ON MONTHLY BASIS	90991
PERITONEAL DIALYSIS TRAINING AND/OR COUNSELING	90992
SUPERVISION OF CHRONIC AMBULATORY PERITONEAL DIALYSIS (CAPD), HOME OR OUT-PATIENT (MONTHLY)	90994
UNLISTED DIALYSIS PROCEDURE, INPATIENT OR OUTPATIENT	90999

HOME VISIT FOR HEMODIALYSIS	99512
HOME INFUSION OF PERITONEAL DIALYSIS, PER VISIT	99559
TRAVEL TIME FOR HOME DIALYSIS EQUIPMENT REPAIR PER MILE	A4830
LABOR CHARGES FOR HOME DIALYSIS EQUIPMENT REPAIR PER HOUR	A4840
PLUMBING AND/OR ELECTRICAL WORK FOR HOME HEMODIALYSIS EQUIPMENT	A4870
END STAGE RENAL DISEASE (ESRD) RELATED SERVICES FOR HOME DIALYSIS PATIENTS PER FULL MONTH; FOR PATIENTS UNDER TWO YEARS OF AGE TO INCLUDE MONITORING FOR ADEQUACY OF NUTRITION, ASSESSMENT OF GROWTH AND DEVELOPMENT, AND COUNSELING OF PARENTS	G0320
END STAGE RENAL DISEASE (ESRD) RELATED SERVICES FOR HOME DIALYSIS PATIENTS PER FULL MONTH; FOR PATIENTS TWO TO ELEVEN YEARS OF AGE TO INCLUDE MONITORING FOR ADEQUACY OF NUTRITION, ASSESSMENT OF GROWTH AND DEVELOPMENT, AND COUNSELING OF PARENTS	G0321
END STAGE RENAL DISEASE (ESRD) RELATED SERVICES FOR HOME DIALYSIS PATIENTS PER FULL MONTH; FOR PATIENTS TWELVE TO NINETEEN YEARS OF AGE TO INCLUDE MONITORING FOR ADEQUACY OF NUTRITION, ASSESSMENT OF GROWTH AND DEVELOPMENT, AND COUNSELING OF PARENTS	G0322
END STAGE RENAL DISEASE (ESRD) RELATED SERVICES FOR HOME DIALYSIS PATIENTS PER FULL MONTH; FOR PATIENTS TWENTY YEARS OF AGE AND OLDER	G0323
END STAGE RENAL DISEASE (ESRD) RELATED SERVICES FOR HOME DIALYSIS (LESS THAN FULL MONTH), PER DAY; FOR PATIENTS UNDER TWO YEARS OF AGE	G0324
END STAGE RENAL DISEASE (ESRD) RELATED SERVICES FOR HOME DIALYSIS (LESS THAN FULL MONTH), PER DAY; FOR PATIENTS BETWEEN TWO AND ELEVEN YEARS OF AGE	G0325
END STAGE RENAL DISEASE (ESRD) RELATED SERVICES FOR HOME DIALYSIS (LESS THAN FULL MONTH), PER DAY; FOR PATIENTS BETWEEN TWELVE AND NINETEEN YEARS OF AGE	G0326
END STAGE RENAL DISEASE (ESRD) RELATED SERVICES FOR HOME DIALYSIS (LESS THAN FULL MONTH), PER DAY; FOR PATIENTS TWENTY YEARS OF AGE AND OVER	G0327
MONTHLY MAINTENANCE CARE FOR PATIENT PERFORMING SELF-HEMO- DIALYSIS AT HOME, PER DAY (NON-STAFF ASSISTED)	M0964
MONTHLY MAINTENANCE CARE FOR PATIENT PERFORMING SELF-PERI- TONEAL DIALYSIS AT HOME, PER DAY (NON-STAFF ASSISTED)	M0968
MONTHLY MAINTENANCE CARE FOR CAPD PATIENT, HOME PATIENT, WITH INTERMITTENT DIALYSIS PERFORMED IN A FACILITY	M0972
SELF DIALYSIS TRAINING, ANY MODE, COMPLETED COURSE	M0974
SELF DIALYSIS TRAINING, ANY MODE, COURSE NOT COMPLETED, PER TRAINING SESSION	M0978
SELF-DIALYSIS, RETRAINING, ANY MODE, PER TRAINING SESSION	M0982
CONTINUOUS AMBULATORY PERITONEAL DIALYSIS (CAPD) TRAINING, COMPLETED COURSE FOR PATIENT NOT PREVIOUSLY TRAINED ON SELF- DIALYSIS MACHINE	M0987
CONTINUOUS AMBULATORY PERITONEAL DIALYSIS (CAPD), RETRAINING, PER TRAINING DAY	M0992
HOME THERAPY, HEMODIALYSIS; ADMINISTRATIVE SERVICES, PROFESSIONAL PHARMACY SERVICES, CARE COORDINATION, AND ALL NECESSARY SUPPLIES AND EQUIPMENT (DRUGS AND NURSING SERVICES CODED SEPARATELY), PER DIEM	S9335
HOME THERAPY, HEMODIALYSIS; ADMINISTRATIVE SERVICES, PROFESSIONAL PHARMACY SERVICES, CARE COORDINATION, AND ALL NECESSARY SUPPLIES AND EQUIPMENT (DRUGS AND NURSING SERVICES CODED SEPARATELY), PER DIEM	S9335
HOME THERAPY; PERITONEAL DIALYSIS, ADMINISTRATIVE SERVICES, PROFESSIONAL PHARMACY SERVICES, CARE COORDINATION AND ALL NECESSARY SUPPLIES AND EQUIPMENT (DRUGS AND NURSING VISITS CODED SEPARATELY), PER DIEM	S9339

CPT= Current Procedural Terminology, HCPCS= Healthcare Common Procedure Coding System, ESRD= End Stage Renal Disease, CAPD = Chronic/Continuous Ambulatory Peritoneal Dialysis

Table 4. Dialysis: Stop Codes

STOP CODE NAME	STOP CODE
ASSISTED HEMODIALYSIS	602
LIMITED SELF CARE HEMODIALYSIS	603
HOME/SELF HEMODIALYSIS TRAINING	604
CAPD (CONTINUOUS AMBULATORY PERITONEAL DIALYSIS)	606
LIMITED SELF CARE CAPD	607
HOME/SELF CAPD TRAINING	608
TELEPHONE/DIALYSIS	609
CONTRACT DIALYSIS	610
TELEPHONE/DIALYSIS	611

C. Clinical Concept: Exclusion Conditions

Rationale: Exclusion criteria were implemented to remove hospitalizations with alternative explanations for anemia that may require RBC transfusions. This study identifies TRAs

specifically, and other causes of anemia or transfusion are not of interest. Specific grounds for exclusion include alternative causes of anemia, such as hemorrhage and alternative causes of transfusion such as surgery. The lists of codes below go into greater detail about the specifics of exclusion criteria. In addition, the following were excluded:

A. *GI bleed:* We defined GI Hemorrhage using IV PPI (a common treatment for GI bleed) and positive guaiac tests (a test for the presence of GI bleed) during the hospital stay and 3 weeks prior to hospitalization.
    a. *IV PPI*: Hospitalizations with an IV administered OrderableItem with a name containing the terms "Lansoprazole", "Omeprazole", "Pantoprazole", "Rabeprazole", or "Esomeprazole" between three weeks prior to admission and the day of discharge (inclusive) were excluded from the study.
    b. *Positive guaiac test*: Hospitalizations with a positive guaiac test between three weeks prior to admission and the day of discharge (inclusive) were excluded. Guaiac tests were defined using clinical review of laboratory test names, topographies, and results. Guaiac tests were identified by laboratory test names containing "FOBT" or ["Occult" and "Blood"] or ["OCC" and "BLD"]. As the guaiac test requires a stool sample, topography's containing the terms "stool", "fecal", or "feces" was also required. A positive result was indicated by the "Abnormal" variable, or where [the lab result value was like "p%" but not like "pending%"], or [where the lab result value was one of the following: "1", "14", "111", "11515"].
    c. *ICD9 diagnosis:* Hemorrhage-specific ICD9 diagnoses are also excluded.
B. *Surgeries*: Hospitalizations with surgery package records of a surgery between 1 day prior to admission and discharge (inclusive) were excluded.
C. Other conditions
    a. *Other ICD9 Diagnosis*: Numerous conditions, which were grounds for exclusion, were identified using HCUP CCS and ICD9 diagnosis codes. See below tables for details.
    b. *Antineoplastic drugs*.

Tables:

1. CDW Patient Lab Chemistry (Guaiac)
2. CDW IV Package (PPI)
3. CDW Surgery Package Surgeries
4. CMS Medicare Part D (Antineoplastic)
5. DSS Pharmacy (Antineoplastic)
6. CDW Inpatient Discharge Diagnosis
7. CDW Outpatient Diagnosis
8. Fee-basis Service Provided Diagnosis
9. Fee-basis Inpatient Invoice Diagnosis

Timeframe:

- Hospitalizations were excluded if ICD9 Diagnosis codes were found between three weeks prior to admission and discharge or if ICD9 diagnosis v07.39 (Other Prophylactic Chemotherapy) occurred between admission and discharge.
- Hospitalizations with the below HCUP CCS Single Level Diagnoses between admission date and discharge date were excluded from the study. Each HCUP CCS classifier is associated with one or more ICD9 diagnosis codes, which represent grounds for exclusion.
- Exclusion criteria were extended to three weeks prior to hospitalization for CCS Single Level Categories 60-64, 153, and 210.

- Hospitalizations with the below VA Drug Class Codes (for antineoplastics) were excluded if they occurred between the time period of 3 months prior to admission, and admission.

Vocabulary: ICD9 procedure codes, HCUP/CCS codes, VA drug class codes (antineoplastics)

Table 5. Exclusion Criteria: ICD9 Diagnosis Codes

ICD9 DESCRIPTION	ICD9 CODE
IRON DEFICIENCY ANEMIA SECONDARY TO BLOOD LOSS (CHRONIC)	280.0
AUTOIMMUNE HEMOLYTIC ANEMIAS	283.0
AUTOIMMUNE HEMOLYTIC DISEASE (COLD TYPE) (WARM TYPE)	283.00
CHRONIC COLD HEMAGGLUTININ DISEASE	283.01
COLD AGGLUTININ DISEASE OR HEMOGLOBINURIA	283.02
HEMOLYTIC ANEMIA, COLD TYPE (SECONDARY) (SYMPTOMATIC)	283.03
HEMOLYTIC ANEMIA, DRUG INDUCED	283.04
HEMOLYTIC ANEMIA, WARD TYPE (SECONDARY) (SYMPTOMATIC)	283.05
AUTOIMMUNE HEMOLYTIC ANEMIAS, OTHER AND UNSPECIFIED	283.09
NON-AUTOIMMUNE HEMOLYTIC ANEMIAS	283.1
NON-AUTOIMMUNE HEMOLYTIC ANEMIA, UNSPECIFIED	283.10
HEMOLYTIC UREMIC SYNDROME	283.11
HEMOLYTIC ANEMIA, TOXIC	283.12
HEMOLYTIC-UREMIC SYNDROME	283.13
OTHER NON-AUTOIMMUNE HEMOLYTIC ANEMIAS	283.19
HEMOGLOBINURIA DUE TO HEMOLYSIS FROM EXTERNAL CAUSES	283.2
ACUTE INTRAVASCULAR HEMOLYSIS	283.20
HEMOGLOBINURIA FROM EXERTION	283.21
HEMOGLOBINLURIA, MARCH	283.22
HEMOGLOBINURIA, PAROXYSMAL (COLD) (NOCTURNAL)	283.23
HEMOGLOBINURIA, DUE TO OTHER HEMOLYSIS	283.24
MARCHIAFAVA-MICHELI SYNDROME	283.25
HEMOGLOBINURIA DUE TO HEMOLYSIS FROM EXTERNAL CAUSES, OTHER AND UNSPECIFIED	283.29
ACQUIRED HEMOLYTIC ANEMIA, UNSPECIFIED	283.9
CHRONIC IDIOPATHIC HEMOLYTIC ANEMIA	283.90
ACQUIRED HEMOLYTIC ANEMIA, NOS	283.99
ACUTE POSTHEMORRHAGIC ANEMIA	285.1
HEMORRHAGE, UNSPECIFIED	459.0
ACUTE GASTRITIS WITH HEMORRHAGE	535.01
ATROPHIC GASTRITIS W HEMORRHAGE	535.11
ALCOHOLIC GASTRITIS W HEMORRHAGE	535.31
OTHER SPECIFIED GASTRITIS W HEMORRHAGE	535.41
UNSPECIFIED GASTRITIS AND GASTRODUODENITIS W HEMORRHAGE	535.51
DUODENITIS WITH HEMORRHAGE	535.61
ANGIODYSPLASIA OF STOMACH AND DUODENUM W HEMORRHAGE	537.83
DIVERTICULOSIS OF COLON W HEMORRHAGE	562.12
DIVERTICULITIS OF COLON W HEMORRHAGE	562.13
PERFORATION OF GALLBLADDER	575.4
HEMORRHAGE INTO BLADDER WALL	596.7
HEMATURIA	599.7
HEMATURIA, UNSPECIFIED	599.70
GROSS HEMATURIA	599.71
MICROSCOPIC HEMATURIA	599.72
LUPUS ERYTHEMATOSUS	695.4
HEMOPTYSIS	786.3
HEMOPTYSIS, UNSPECIFIED	786.30

ICD9=International Classification of Diseases 9

Table 6. Exclusion Criteria: HCUP CCS Codes

CONDITION	LABEL	HCUP CODE
MALIGNANCY	CANCER OF HEAD AND NECK	11
MALIGNANCY	CANCER OF ESOPHAGUS	12
MALIGNANCY	CANCER OF STOMACH	13
MALIGNANCY	CANCER OF COLON	14
MALIGNANCY	CANCER OF RECTUM AND ANUS	15
MALIGNANCY	CANCER OF LIVER AND INTRAHEPATIC BILE DUCT	16
MALIGNANCY	CANCER OF PANCREAS	17
MALIGNANCY	CANCER OF OTHER GI ORGANS; PERITONEUM	18
MALIGNANCY	CANCER OF BRONCHUS; LUNG	19
MALIGNANCY	CANCER; OTHER RESPIRATORY AND INTRATHORACIC	20
MALIGNANCY	CANCER OF BONE AND CONNECTIVE TISSUE	21
MALIGNANCY	MELANOMAS OF SKIN	22
MALIGNANCY	OTHER NON-EPITHELIAL CANCER OF SKIN	23
MALIGNANCY	CANCER OF BREAST	24

MALIGNANCY	CANCER OF UTERUS	25
MALIGNANCY	CANCER OF CERVIX	26
MALIGNANCY	CANCER OF OVARY	27
MALIGNANCY	CANCER OF OTHER FEMALE GENITAL ORGANS	28
MALIGNANCY	CANCER OF PROSTATE	29
MALIGNANCY	CANCER OF TESTIS	30
MALIGNANCY	CANCER OF OTHER MALE GENITAL ORGANS	31
MALIGNANCY	CANCER OF BLADDER	32
MALIGNANCY	CANCER OF KIDNEY AND RENAL PELVIS	33
MALIGNANCY	CANCER OF OTHER URINARY ORGANS	34
MALIGNANCY	CANCER OF BRAIN AND NERVOUS SYSTEM	35
MALIGNANCY	CANCER OF THYROID	36
MALIGNANCY	HODGKIN‘S DISEASE	37
MALIGNANCY	NON-HODGKIN‘S LYMPHOMA	38
MALIGNANCY	LEUKEMIAS	39
MALIGNANCY	MULTIPLE MYELOMA	40
MALIGNANCY	CANCER; OTHER AND UNSPECIFIED PRIMARY	41
MALIGNANCY	SECONDARY MALIGNANCIES	42
MALIGNANCY	MALIGNANT NEOPLASM WITHOUT SPECIFICATION OF SITE	43
MALIGNANCY	NEOPLASMS OF UNSPECIFIED NATURE OR UNCERTAIN BEHAVIOR	44
MALIGNANCY	MAINTENANCE CHEMOTHERAPY; RADIOTHERAPY	45
MALIGNANCY	BENIGN NEOPLASM OF UTERUS	46
MALIGNANCY	OTHER AND UNSPECIFIED BENIGN NEOPLASM	47
HEMATOLOGICAL	ACUTE POSTHEMORRHAGIC ANEMIA	60
HEMATOLOGICAL	SICKLE CELL ANEMIA	61
HEMATOLOGICAL	COAGULATION AND HEMORRHAGIC DISORDERS	62
HEMATOLOGICAL	DISEASES OF WHITE BLOOD CELLS	63
HEMATOLOGICAL	OTHER HEMATOLOGIC CONDITIONS	64
GI BLEED	GASTROINTESTINAL HEMORRHAGE	153
GI BLEED	NONINFECTIOUS GASTROENTERITIS	154
GI BLEED	OTHER GASTROINTESTINAL DISORDERS	155
LUPUS	SYSTEMIC LUPUS ERYTHEMATOSUS AND CONNECTIVE TISSUE DISORDERS	210
INJURY	FRACTURE OF NECK OF FEMUR (HIP)	226
INJURY	SPINAL CORD INJURY	227
INJURY	SKULL AND FACE FRACTURES	228
INJURY	FRACTURE OF UPPER LIMB	229
INJURY	FRACTURE OF LOWER LIMB	230
INJURY	OTHER FRACTURES	231
INJURY	SPRAINS AND STRAINS	232
INJURY	INTRACRANIAL INJURY	233
INJURY	CRUSHING INJURY OR INTERNAL INJURY	234
INJURY	OPEN WOUNDS OF HEAD; NECK; AND TRUNK	235
INJURY	OPEN WOUNDS OF EXTREMITIES	236
INJURY	COMPLICATION OF DEVICE; IMPLANT OR GRAFT	237
INJURY	COMPLICATIONS OF SURGICAL PROCEDURES OR MEDICAL CARE	238
INJURY	SUPERFICIAL INJURY; CONTUSION	239
INJURY	E CODES: CUT/PIERCEB	2601
INJURY	E CODES: DROWNING/SUBMERSION	2602
INJURY	E CODES: FALL	2603
INJURY	E CODES: FIRE/BURN	2604
INJURY	E CODES: FIREARM	2605
INJURY	E CODES: MACHINERY	2606
INJURY	E CODES: MOTOR VEHICLE TRAFFIC (MVT)	2607
INJURY	E CODES: PEDAL CYCLIST; NOT MVT	2608
INJURY	E CODES: PEDESTRIAN; NOT MVT	2609
INJURY	E CODES: TRANSPORT; NOT MVT	2610
INJURY	E CODES: NATURAL/ENVIRONMENT	2611
INJURY	E CODES: OVEREXERTION	2612
INJURY	E CODES: POISONING	2613
INJURY	E CODES: STRUCK BY; AGAINST	2614
INJURY	E CODES: SUFFOCATION	2615
INJURY	E CODES: ADVERSE EFFECTS OF MEDICAL CARE	2616
INJURY	E CODES: ADVERSE EFFECTS OF MEDICAL DRUGS	2617
INJURY	E CODES: OTHER SPECIFIED AND CLASSIFIABLE	2618
INJURY	E CODES: OTHER SPECIFIED; NEC	2619
INJURY	E CODES: UNSPECIFIED	2620

INJURY	E CODES: PLACE OF OCCURRENCE	2621

HCUP CCS = Healthcare Cost and Utilization Project Clinical Classifications Software

Table 7. Exclusion Criteria: VA Drug Class Codes (Antineoplastics)

DRUG CLASSIFICATION	VA DRUG CLASS CODE
ANTINEOPLASTICS	AN000
ANTINEOPLASTICS,ALKYLATING AGENTS	AN100
ANTINEOPLASTIC ANTIBIOTICS	AN200
ANTINEOPLASTICS,ANTIMETABOLITES	AN300
ANTINEOPLASTIC ADJUVANTS	AN400
ANTINEOPLASTIC HORMONES	AN500
ANTINEOPLASTIC RADIOPHARMACEUTICALS	AN600
PROTECTIVE AGENTS	AN700
ANTINEOPLASTIC,OTHER	AN900

VA = Veterans Affairs

D. Clinical concept: Anemia

Rationale: Patients with evidence of anemia related to ESRD were identified in this study as a clinical marker for a potential TRA. Evidence of anemia was determined by LOINC and laboratory values, which were validated during abstraction of the medical record. These fields were leveraged in the clinical algorithm as the data definition of anemia.

Anemia was defined as hemoglobin (HGB) < 9.0 mg/dL. If Hemoglobin was not reported, 1/3 of hematocrit (HCT) (<27%) was substituted. Defining Anemia through laboratory values required clinical review of aggregate laboratory results. This review began by identifying LOINC codes identified with HGB and HCT, querying all laboratory records associated with those LOINC codes, and then sorting through the associated lab test names and topographies.

After the LOINC codes were queried, and lab test names reviewed, the following logic was used to restrict to HGB and HCT specific tests. Topographies were intended to reflect blood, serum or plasma, and contained any of the following: “bld”, “blood”, “venous”, “arterial”, “erythrocyte”, “plasma”, “serum” and did not contain any of the following: “urine” or “fluid.” Accepted lab test names contained any of the following: “hemoglobin”, “hgb”, “thb”, “hematocrit”, "hct", but did not include any of the following: "pulse oxim", "fhhb", "reticulocyte", "occult."

Tables:

1. CDW Lab Chemistry

Timeframe: Anemia had to occur +/- 24 hours from admission
Vocabulary: LOINC

Table 8. Clinical Anemia: LOINC codes used to identify Hemoglobin (HGB) and Hematocrit (HCT)

COMPONENT	LOINC
HEMATOCRIT	11151-8, 11153-4, 11271-4, 13508-7, 17809-5, 20570-8, 30398-2, 31100-1, 32354-3, 41654-5, 41655-2, 41986-1, 39227-4, 42908-4, 43416-7, 4544-3, 4545-0, 47640-8, 48703-3, 55781-9, 62241-5, 70168-0, 70169-8, 71828-8, 71829-6, 71830-4, 71831-2, 71832-0, 71833-8
HEMOGLOBIN	14775-1, 30313-1, 30350-3, 30351-1, 30352-9, 30353-7, 30354-5, 33025-8, 33026-6, 33509-1, 33517-4, 34618-9, 35183-3, 40719-7, 42243-6, 42810-2, 48035-0, 49137-3, 50559-4, 54289-4, 55782-7, 57751-0, 5794-3, 59260-0, 61180-6, 69950-4, 71694-4, 717-9, 718-7, 719-5, 722-9, 723-7, 724-5, 725-2, 726-0, 73895-5, 76769-9, 20509-6
HEMATOCRIT/HEMOGLOBIN	16931-8
HEMOGLOBIN & HEMATOCRIT PANEL	24360-0

LOINC= *Logical Observation Identifiers Names and Codes*

E. Clinical Concept: RBC Transfusion

Rationale: Evidence of a RBC transfusion during admission with concurrent anemia related to ESRD were identified in this study as a clinical marker for a potential TRA. The ideal source of blood bank records available within the VHA is the VistA Blood Establishment Computer Software (VBECS). However, due to FDA’s regulatory status of VBECS as a

510(k) device; the CDW does not maintain a mirror copy for research. To bolster our RBC transfusion detection, we explored the Managerial Cost Accounting (MCA) system, an alternative domain that is mirrored in the CDW, to investigate blood product administration. For these reasons, we used multiple data sources to identify RBC transfusions: ICD9 procedure codes, CPT/HCPCS procedure codes, VHA orderable items, and MCA feeder keys. As part of our preliminary consultations; clinicians indicated that relevant RBC transfusions were likely administered promptly within 24 hours of admission. Records of RBC transfusions found in the VHA data benefit from associated administration timestamps and allowed us to account for the time a transfusion was given.

Tables:

1. CDW Inpatient ICD9 Procedures
2. CDW Inpatient CPT Procedures
3. CDW Outpatient CPT Procedures
4. MCA LAB
5. MCA ECS
6. CDW CPRS Orders

Time Frame: RBC Transfusion occurred after anemia, but in the first 24 hours of admission. Transfusion orders were also allowed to occur prior to admission but after anemia.

Vocabulary: ICD9 procedure codes, CPT/HCPCS codes, orderable items, MCA feeder keys

RBC Transfusion Identification using CDW ICD9 and CPT

ICD9 Procedures

The below ICD9 procedures were considered evidence of RBC transfusions. These records were found in the CDW inpatient data domain.

Table 9. Clinical RBC Transfusion: ICD9 Procedure codes used to identify RBC Transfusions

ICD9 PROCEDURE DESCRIPTION	ICD9 PROCEDURE CODE
OTHER TRANSFUSION OF WHOLE BLOOD	99.03
TRANSFUSION OF PACKED CELLS	99.04
ICD9=International Classification of Diseases 9	

CPT/HCPCS Procedures

The below CPT/HCPCS codes were considered evidence of RBC transfusion. These records were found in the CDW inpatient and outpatient data domains. Note that in VHA, outpatient procedures may occur during inpatient stays (prior to discharge).

Table 10. Clinical RBC Transfusion: CPT/HCPCS Procedure codes used to identify RBC Transfusions

CPT DESCRIPTION	CPT CODE
TRANSFUSION, BLOOD OR BLOOD COMPONENTS	36430
BLOOD (WHOLE), FOR TRANSFUSION, PER UNIT	P9010
BLOOD, SPLIT UNIT	P9011
RED BLOOD CELLS, LEUKOCYTES REDUCED, EACH UNIT	P9016
RED BLOOD CELLS, EACH UNIT	P9021
RED BLOOD CELLS, WASHED, EACH UNIT	P9022
RED BLOOD CELLS, IRRADIATED, EACH UNIT	P9038
RED BLOOD CELLS, DEGLYCEROLIZED, EACH UNIT	P9039
RED BLOOD CELLS, LEUKOCYTES REDUCED, IRRADIATED, EACH UNIT	P9040
WHOLE BLOOD OR RED BLOOD CELLS, LEUKOCYTES REDUCED, CMV-NEGATIVE	P9051
WHOLE BLOOD OR RED BLOOD CELLS, LEUKOCYTES REDUCED, FROZEN,	P9054
WHOLE BLOOD, LEUKOCYTES REDUCED, IRRADIATED, EACH UNIT	P9056
RED BLOOD CELLS, LEUKOCYTES REDUCED, CMV-NEGATIVE, IRRADIATE	P9058

CPT= Current Procedural Terminology, HCPCS = Healthcare Common Procedure Coding System, RBC = Red Blood Cell, CMV = *Cytomegalovirus*

Orderable Items

The below Orderable Items were considered evidence of RBC transfusion. These codes represent orders for RBC transfusions, regardless of whether they were carried out. Chart review revealed that inclusion of these codes increased sensitivity to RBC transfusions, but decreased specificity. Orderable Items were not allowed to have associated status of: "pending", "cancelled", "unreleased", or "expired".

Table 11. Clinical RBC Transfusion: Orderable Items Used as Evidence of RBC Transfusion

ORDERABLE ITEM NAME
BLOOD RELEASE AND TRANSFUSE
BLOOD TRANSFUSION
PACKED RED BLOOD CELL
PACKED RED BLOOD CELLS
PRBC'S TO TRANSFUSE
RBC
RED BLOOD CELLS
TRANSFUSE PACKED RBC
TRANSFUSE RED BLOOD CELLS

RBC = Red Blood Cell

RBC Transfusion Identification using MCA

Background: MCA

VA's cost allocation system, the Decision Support System (DSS), now known as the MCA system, generates cost data on VA hospital stays and health care encounters using programs that run on pre-existing VA relational databases to provide information to managers and physicians. Cost of intermediate products, such as units of blood, are evaluated by MCA programs.[1] MCA collects data in the outpatient and inpatient settings.[2] Tables within the MCA domain were used because the DSS application software inherently compares records to VBECS for use in the Laboratory Blood Bank (LBB) Comparative Report. The LBB compares information from the VBECS DSS EXTRACT file with blood bank records reported to DSS.[3]

We examined two of the many MCA tables to identify RBC transfusions: the event capture system (ECS) and Laboratory (LAB) tables.[1] The LAB table contains information on a patient's laboratory test .[4]

MCA feeder keys in these tables which contain information about the product extracted by MCA.[3] We found that a few of the feeder keys identified as records of RBC transfusion administration showed a high degree of correlation with the blood product key found in CAPRI, which provides read access to the blood bank data.

Selection of MCA Feeder Keys representing RBC transfusions

To identify potentially relevant feeder keys, data from the ECS and LAB tables were queried within one day prior to admission, and two days after admission. Records with procedure or test names containing "transfus", "Red Blood Cell", "RBC", "blood product", "blood bank", and "VBECS" were selected for review. Also reviewed were cases where the feeder key or associated name contained a CPT code identified previously as evidence of RBC transfusion. A clinician reviewed the feeder keys and corresponding procedure/test names. In the event of

questionable procedure/test names, a manual review of patient records with the respective feeder key was performed using CAPRI. This allowed us to view VBECS records and the associated blood product keys to determine if a questionable feeder key was representative of a RBC transfusion. Feeder keys associated with a RBC transfusion and approved by clinician review were included in our code set for the identification of RBC transfusion (Table 12).

We determined that feeder keys associated with procedure/test names containing terms related to “ABO”, “Count”, “Fresh Frozen Plasma”, “Folate”, “Indices”, “Lysate” “Panel”, “RBA”, “Request”, and “Saline” were not representative of a RBC transfusion. Some of these terms indicate the wrong concept (e.g. fresh frozen plasma), while others indicate the wrong topography (e.g. Urine), and others indicate a type and cross (e.g. ABO). Type and cross records were not included because they did not always indicate the blood product was administered and did not provide a consistent record of RBC transfusions. Some of these terms were added as a result of clinician review.

Table 12. Clinical RBC Transfusion: MCA Feeder Keys Used as Evidence of RBC Transfusion

FEEDER KEY	TEST NAME*	FEEDER KEY	TEST NAME*
336	E0336V00 AS-1/RBC/500ML/L	PRBC	RBC LEUKOCYTES REDUCED
4741	AS-3/RBC/PHER-1/LR	PRBC	RED BLOOD CELLS
4761	AS-3/RBC/PHER-2/LR	PRC1	RED BLOOD CELLS
4771	AS-3/RBC/PHER-1/LR/ACD-A	PRC2	RED BLOOD CELLS
5RBC	5RBC	PRC3	RED BLOOD CELLS
93960	VBECS	R1	RBC-LEUK POOR NON-IRRADIA
A1LR	RBC LEUKOCYTES REDUCED	R1/L	LEUKOREDUCED PRBC\61264
A3L2	AS-3 RBC 2 PHERESED,LR	R1/L	RED BLOOD CELLS
A3LI	AS-3 RBC LEUKOCYTES REDUC	R101	AS1 RBC LR
A3LK	RBC, LEUKOREDUCED	R2/L	AS-1 RBC, LEUKO REDUCT
A3LR	AS-3 RED BLOOD CELLS LEUK	R201	AS2 RBC LR
A3LR	RBC LEUKOCYTES REDUCED	R501	AS5 RBC LR
A3P1	AS-3 RBC LEUKORED (PH BAG	RA/L	RA/L-RBC, LEUKO REDUCED
A3PL	AS3 RBC,1PHERESED LREDUCE	RA1	CPDA1 RBC
ALRC	AS-1 RBC, LEUKO REDUCED	RA1	CPDA-1 RED BLOOD CELLS
ARLA	RBC LEUKOCYTES REDUCED	RA1L	RBC AS1 LEUKO 450
AS 3	RED BLOOD CELLS	RA34	RBC LEUKO-REDUCED
AS-1	AS-1 RBC LEUKOCYTES REDUC	RAA	RED BLOOD CELLS
AS-1	AS-1 RED BLOOD CELLS	RAI	RED BLOOD CELLS
AS-1	RBC LEUKOCYTES REDUCED	RBC	*RED BLOOD CELLS
AS-3	AS-3 RED BLOOD CELLS, FIL	RBC	81759-RBC
AS-3	RED BLOOD CELLS	RBC	86799 RED BLOOD CELLS
AS-5	AS-5 AS 5 RED BLOOD CELL	RBC	86802 RBC (VARIOUS FORMS)
AS-5	AS-5 RED BLOOD CELLS	RBC	AS-1 LEUKO RBC
AS-5	PRBC	RBC	PACKED RED BLOOD CELLS
AS-5	RBC	RBC	RBC
AS-5	RED BLOOD CELL LEUKO REDU	RBC	RBC CELLS, RED BLOOD

AS-5	RED BLOOD CELLS UNIT	RBC	RBC CPD>AS1 LUK
AS/I	RBC LEUKOCYTES REDUCED	RBC	RBC CPD>AS1 LUKOPR
AS/I	RED BLOOD CELLS IRRIDATED	RBC	RBC DEGLYCEROLIZED UNIT
AS\3	AS-3 RBC LEUKO-REDUCED	RBC	RBC IRAD,LEUKO-RED CPDA-1
AS_1	AS-1 RED BLOOD CELLS	RBC	RBC LEUKOCYTES REDUC
AS1E	AS-1 LEUKO RBC	RBC	RBC LEUKOCYTES REDUCED
AS1I	RBC LEUKOCYTES REDUCED	RBC	RBC LEUKOREDUCED
AS1L	AS1 RBC LEUKOCYTE FLTRED	RBC	RBC RED BLOOD CEL
AS1L	AS1 RBC,LEUKOCYTE FLTRED	RBC	RBC RED BLOOD CEL 61263
AS1L	RBC, LEUKO-REMOVED	RBC	RBC REJUVENATED UNIT
AS1L	RED BLOOD CELLS	RBC	RBC,ANY
AS1N	AS-1 NONLEUKOREDUCED RBC	RBC	RBC/RBC LEUKO REDUCED
AS3	AS-3 RBC PHER	RBC	RBC/RBC LEUKOCYTE REDUCED
AS3	PACKED RED BLOOD CELLS	RBC	RBC/RBC LEUKOCYTES REDUCE
AS3I	RBC LEUKOCYTES REDUCED	RBC	RBC-PACKED RBC FILTERED
AS3L	AS-3 RBCLR	RBC	RBC-RBC
AS3L	RBC, LEUKOREDUCED	RBC	RBC-RED BLOOD CELLS
AS3P	AS3P-RBC CP2D/5	RBC	RED BLOOD CELL
AS5	RBC AS-5	RBC	RED BLOOD CELLS
ASFL	AS-1 RED BLOOD CELLS,LEUK	RBC	RED BLOOD CELLS - RASI
ASLD	RBC LEUKOCYTES REDUCED	RBC	RED BLOOD CELLS (RBC)
ASRD	AS-1 RBC LEUKO-REDUCED	RBC	RED BLOOD CELLS LEUKO
CMF	CMF-PACKED RBC FILTERED	RBC	RED BLOOD CELLS LEUKOREDU
CP1	RED BLOOD CELLS LEUK REDU	RBC	RED BLOOD CELLS UNIT
CPDA	CPDA-1 RBCS (4 OR MOR	RBC	RED BLOOD CELLS WASHED
CPDA	RBC CPDA-1	RBC	RED BLOOD CELLS, IRRAD AC
ERBC	RBC LEUKOCYTE REDUC&IRRAD	RBC	VBECS RED BLOOD CELL
FIL	AS-1 RED BLOOD CELLS,FILT	RBC	VBECS RED BLOOD CELLS
I_RBC	86799 RED BLOOD CELLS	RBC	WASHED PRBC'S
I_RBC	I_RBC/IRRADIATED UNIT	RBC	ZRBC
I_RBC	IRRAD RED BLOOD CELLS	RBC	ZZECS RED BLOOD CELL
I_RBC	RBC	RBC	ZZECS RED BLOOD CELLS
IAS3	RBC LEUKOCYTES REDUCED	RBC	ZZRBC
ILRR	IRRAD, LEUKOREDUCED RBC	RBC	ZZWASHED PRBC'S
IRL3	AS-3 RBC IRR-LP	RBC\	RBC\
L_RBC	RED BLOOD CELLS	RBC1	RED BLOOD CELLS
LA1	RBC LEUKOCYTES REDUCED	RBC5	RED BLOOD CELLS
LA1	RED BLOOD CELLS LEUKOCYTE	RBCL	RBCL-RBC LEUKOCYTE

			REDUCE
LA22	86802 RBC LEUKOCYTES RED	RBCN	ISBT RBC CPDA-1
LARC	RBC LEUKOCYTES REDUCED	RBCP	RBCP PACKED RED CELLS
LCA4	LUEKO REDUCED RBC	RC/F	RED BLOOD CELLS,LEUK REDU
LCP1	RBC LEUKOCYTES REDUCED	RC/L	CPD RED BLOOD CELLS,LEUK
LCP2	RBC LEUKOCYTES REDUCED	RC2	RED BLOOD CELLS LEUKOCYTE
LCP3	RBC LEUKOCYTES REDUCED	RC32	RED BLOOD CELLS LEUKOCYTE
LCPA	RBC CPDA1 LEUKO	RC5	AS-5 RED BLOOD CELLS
LCR1	RBC LEUKOCYTES REDUCED	RC5	RED BLOOD CELLS LEUKOCYTE
LCRC	RBC LEUKOCYTES REDUCED	RC6	RED BLOOD CELLS LEUKOCYTE
LR61	RBC LEUKOCYTES REDUCED	RCFD	RED BLOOD CELLS,LEUK REDU
LRA3	86802 RBC LEUKOCYTES RED	RCL	RBC LEUKOCYTES REDUCED
LRB5	LEUKOREDUCED AS-5 RBC	RCLD	RED BLOOD CELLS LEUKOCYTE
LRBC	RBC LEUKOCYTES REDUCED	RCLP	RED BLOOD CELLS LEUK RED
LRBC	RBC, LEUKOCYTES REDUCED	RCP2	AS-1 RBC PHER BAG #2
LRC	RBC LEUKOCYTES REDUCED	RIL	RBC LEUKOCYTES REDUCED
LRC1	RBC LEUKOCYTES REDUCED	RILR	AS1 RBC IRRAD LR (CPD)
LRC2	RBC LEUKOCYTES REDUCED	RLA1	RBC LEUKOCYTES REDUCED
LRC3	AS-3 LEUKOREDUCED PRBC	RLR	RBC LEUKOCYTES REDUCED
LRF	PACKED RED BLOOD CELLS	RLR2	RLR2-RBC, LEUKO REDUCED
LRPC	AS-1 RBC, LEUKOREDUCED	RLRL	AS-3 RBC LEUKOCYTES REDUC
LRPC	RBC LEUKOCYTES REDUCED	RPL2	AS1 RBC 2PHERESED LR ACDA
LRRA	APHERSIS RBC LEUKOCYTES	RPLA	AS1 RBC 1PHERESED LR ACDA
LRRC	RBC LEUKO REDUCED	RRCL	RED BLOOD CELLS LEUKORED
PC	RED BLOOD CELLS	RS1L	RS1L PACKED RBCS
PC41	AS-1 RBC, LEUKOREDUCED	S_RBC	RBC
PCL	RBC LEUKO REDUCD	W1LR	AS-1 RBC'S, IRRADIATED
PCLR	RED BLOOD CELLS LEUKOCYTE	W3L2	AS-3 RBC LEUKO-REDUCED
PCUA	RED BLOOD CELLS, UNI	WAS5	RED BLOOD CELLS UNIT
PRBC	RBC LEUKOCYTES REDUCED	ZB062L	AS-3 RBC 04741(PHERESIS)
		ZB063L	AS-3 RBC 04761 PHERESIS)

AS = Additive Solution, RBC = Red Blood Cell, LR = LEUKOCYTES REDUCED, IRR-Irradiated, CPD = citrate-phosphate-dextrose, CPDA = citrate-phosphate-dextrose-*adenine*
*Values are reported as they exist in the database, this includes misspelled and unfinished words
*Some acronyms are not listed because they are specific to MCA DSS or VBECS domains

Section 2. Clinical Algorithm TRA

Rationale: The *clinical algorithm (CA)* was created to identify a TRA using data that is not typically available in historical CMS (discussed in Section 3). For our study, this applies to hemoglobin laboratory values, as well as data used in our exclusion criteria which included internal VHA concepts found in the surgical package to identify surgery, IV package to identify Proton Pump Inhibitor administration, and laboratory to identify positive guaiac test results. The *CA* defines a TRA as hospital admission in a population of ESRD (Section 1A) patients who have had dialysis (Section 1B), where there is evidence of anemia (HGB < 9.0 mg/dL or HCT < 27 %) (Section 1D) and a RBC transfusion (Section 1E) in the absence of exclusion criteria (Section 1C). Anemia was required to occur +/- 24 hours before or after admission. Evidence of a RBC transfusion had to occur after anemia and during the first 24 hours of the admission. We allowed RBC transfusion orders (Table 11) to also account for RBC transfusion administration in order to increase sensitivity. Orders for a RBC transfusion were included as evidence of administration due to our inability to access VBECs blood bank database, which contains all records of RBC transfusion administration in the VA.[5] Full definitions are described in sections above.

CAs data definitions described in the sections above:

- CA ESRD (Section 1A)
- CA Anemia (Section 1D)
- CA RBC Transfusion (Section 1E)
- CA Exclusion Criteria (Section 1C)

Section 3. Claims Based TRA

3.1 Claims algorithms rationale, definitions and limitations

Rationale*:* Four Claims Based Algorithms (CBA) were defined and used as exploratory measures, designed to detect TRA/TRA-Primary in claims based data typical of Centers for Medicare and Medicaid Services (CMS) Inpatient Research Identifiable Files using key features also present in VA data. This would provide a framework for those who do not have access to clinical data to apply similar TRA/TRA-primary detection procedures to claims data, using validated algorithms. Note that some exclusion criteria involved data that may not be available in claims data and must be taken into consideration when transporting these algorithms to CMS or commercial databases.

Key Features - VA Data to CMS Inpatient Research Identifiable Files for TRA identification:

- Principal discharge diagnosis code (reason for the admission)
- Secondary diagnosis codes
- Procedure codes
- CMS “present on admission” was not included into CBA because the VHA does not contain a direct equivalent
- RBC transfusions were allowed to occur on the day of admission or the day after admission

Limitations: As described above, the process we used allowed us to mimic information found in the CMS Inpatient Research Identifiable files using VA data. However, there are limitations to the direct application of these algorithms to CMS data. CBAs should be applied

with caution and validation in a CMS or commercial setting. Nevertheless, the rationale and justification are consistent with how one would implement the algorithms. Also, note that we used additional clinical data in our exclusion criteria that may not be contained in other data sets.

Defining CBA components: CBA used various terminology resources to detect the occurrence of TRA/TRA-Primary.
The following definitions are used to define the various CBAs.

- RBC transfusion: A RBC transfusion on date of admission or the following date, as coded in ICD9 (Table 9) or CPT/HCPCS (Table 10) procedure codes, product administration orders (Table 11) or DSS feeder keys (Table 12)
- CKD: a principal or secondary ICD9 discharge diagnosis of CKD (see Table 13 below)
- Anemia: a principal or secondary ICD9 discharge diagnosis of anemia (see Table 14 below)

Tables:

1. CDW Inpatient Discharge Diagnoses

Timeframe:

- CKD Diagnosis – On discharge date
- RBC Transfusion – On date of admission or the following date
- Anemia diagnosis – On discharge date

Vocabulary: ICD9 Diagnosis codes

Table 13: Claims Based Algorithm: ICD9 Inpatient Discharge Diagnoses of CKD, ESRD, or Anemia in CKD

ICD9 CODE	ICD9 DESCRIPTION
285.21	ANEMIA IN CHRONIC KIDNEY DISEASE
403.0	MALIGNANT HYPERTENSIVE RENAL DISEASE
403.00	HYPERTENSIVE CHRONIC KIDNEY DISEASE, MALIGNANT, WITH CHRONIC KIDNEY DISEASE STAGE I THROUGH STAGE IV, OR UNSPECIFIED
403.01	HYPERTENSIVE CHRONIC KIDNEY DISEASE, MALIGNANT, WITH CHRONIC KIDNEY DISEASE STAGE V OR END STAGE RENAL DISEASE
403.1	BENIGN HYPERTENSIVE RENAL DISEASE
403.10	HYPERTENSIVE CHRONIC KIDNEY DISEASE, BENIGN, WITH CHRONIC KIDNEY DISEASE STAGE I THROUGH STAGE IV, OR UNSPECIFIED
403.11	HYPERTENSIVE CHRONIC KIDNEY DISEASE, BENIGN, WITH CHRONIC KIDNEY DISEASE STAGE V OR END STAGE RENAL DISEASE
403.9	UNSPECIFIED HYPERTENSIVE RENAL DISEASE
403.90	HYPERTENSIVE CHRONIC KIDNEY DISEASE, UNSPECIFIED, WITH CHRONIC KIDNEY DISEASE STAGE I THROUGH STAGE IV, OR UNSPECIFIED
403.91	HYPERTENSIVE CHRONIC KIDNEY DISEASE, UNSPECIFIED, WITH CHRONIC KIDNEY DISEASE STAGE V OR END STAGE RENAL DISEASE
404.0	MALIGNANT HYPERTENSIVE HEART AND RENAL DISEASE
404.00	HYPERTENSIVE HEART AND CHRONIC KIDNEY DISEASE, MALIGNANT, WITHOUT HEART FAILURE AND WITH CHRONIC KIDNEY DISEASE STAGE I THROUGH STAGE IV, OR UNSPECIFIED
404.01	HYPERTENSIVE HEART AND CHRONIC KIDNEY DISEASE, MALIGNANT, WITH HEART FAILURE AND WITH CHRONIC KIDNEY DISEASE STAGE I THROUGH STAGE IV, OR UNSPECIFIED
404.02	HYPERTENSIVE HEART AND CHRONIC KIDNEY DISEASE, MALIGNANT, WITHOUT HEART FAILURE AND WITH CHRONIC KIDNEY DISEASE STAGE V OR END STAGE RENAL DISEASE
404.03	HYPERTENSIVE HEART AND CHRONIC KIDNEY DISEASE, MALIGNANT, WITH HEART FAILURE AND WITH CHRONIC KIDNEY DISEASE STAGE V OR END STAGE RENAL DISEASE
404.1	BENIGN HYPERTENSIVE HEART AND RENAL DISEASE
404.10	HYPERTENSIVE HEART AND CHRONIC KIDNEY DISEASE, BENIGN, WITHOUT HEART FAILURE AND WITH CHRONIC KIDNEY DISEASE STAGE I THROUGH STAGE IV, OR UNSPECIFIED
404.11	HYPERTENSIVE HEART AND CHRONIC KIDNEY DISEASE, BENIGN, WITH HEART FAILURE AND WITH CHRONIC KIDNEY DISEASE STAGE I THROUGH STAGE IV, OR UNSPECIFIED
404.12	HYPERTENSIVE HEART AND CHRONIC KIDNEY DISEASE, BENIGN, WITHOUT HEART FAILURE AND WITH CHRONIC KIDNEY DISEASE STAGE V OR END STAGE RENAL DISEASE

404.13	HYPERTENSIVE HEART AND CHRONIC KIDNEY DISEASE, BENIGN, WITH HEART FAILURE AND CHRONIC KIDNEY DISEASE STAGE V OR END STAGE RENAL DISEASE
404.9	UNSPECIFIED HYPERTENSIVE HEART AND RENAL DISEASE
404.90	HYPERTENSIVE HEART AND CHRONIC KIDNEY DISEASE, UNSPECIFIED, WITHOUT HEART FAILURE AND WITH CHRONIC KIDNEY DISEASE STAGE I THROUGH STAGE IV, OR UNSPECIFIED
404.91	HYPERTENSIVE HEART AND CHRONIC KIDNEY DISEASE, UNSPECIFIED, WITH HEART FAILURE AND WITH CHRONIC KIDNEY DISEASE STAGE I THROUGH STAGE IV, OR UNSPECIFIED
404.92	HYPERTENSIVE HEART AND CHRONIC KIDNEY DISEASE, UNSPECIFIED, WITHOUT HEART FAILURE AND WITH CHRONIC KIDNEY DISEASE STAGE V OR END STAGE RENAL DISEASE
404.93	HYPERTENSIVE HEART AND CHRONIC KIDNEY DISEASE, UNSPECIFIED, WITH HEART FAILURE AND CHRONIC KIDNEY DISEASE STAGE V OR END STAGE RENAL DISEASE
585.	CHRONIC RENAL FAILURE
585.1	CHRONIC KIDNEY DISEASE, STAGE I
585.2	CHRONIC KIDNEY DISEASE, STAGE II (MILD)
585.3	CHRONIC KIDNEY DISEASE, STAGE III (MODERATE)
585.4	CHRONIC KIDNEY DISEASE, STAGE IV (SEVERE)
585.5	CHRONIC KIDNEY DISEASE, STAGE V
585.6	END STAGE RENAL DISEASE
585.9	CHRONIC KIDNEY DISEASE, UNSPECIFIED

CKD = Chronic Kidney Disease, ESRD = End Stage Renal Disease, ICD9=International Classification of Diseases 9

Table 14: ICD9 Inpatient Discharge Diagnoses of Anemia

ICD9 CODE	ICD9 DESCRIPTION
285.00	HYPOCHROMIC ANEMIA WITH IRON LOADING
285.01	SIDEROACHRESTIC ANEMIA
285.21	ANEMIA IN CHRONIC KIDNEY DISEASE
285.29	ANEMIA OF OTHER CHRONIC DISEASE
285.8	OTHER SPECIFIED ANEMIAS
285.89	OTHER SPECIFIED ANEMIAS, NOT ELSEWHERE CLASSIFIED
285.9	ANEMIA, UNSPECIFIED
285.90	ESSENTIAL ANEMIA
285.91	NORMOCHROMIC ANEMIA, NOT DUE TO BLOOD LOSS
285.92	PROFOUND ANEMIA
285.93	PROGRESSIVE ANEMIA
285.94	SECONDARY ANEMIA
285.99	ANEMIA, NOT OTHERWISE SPECIFIED

ICD9=International Classification of Diseases 9

3.2 Claims algorithms and Concept Comparisons

Rationale: Different coding practices led us to create four claims algorithms to identify TRA and TRA-primary. Each algorithm was applied to each definition to determine the best identifier in claims data. Claims algorithms are described in Table 15 below.

Table 15. TRA and TRA-Primary concept definitions and four corresponding CBAs designed to identify them in the data

Concept Definition	*Claims Algorithm Variations*
Concept: TRA<br>• A RBC transfusion used to treat ESRD-related anemia on admission regardless of the reason for admission<br><br>*Concept*: TRA-Primary<br>• A RBC transfusion on admission for the primary purpose of treating ESRD-related anemia	1. *CBA 1– Primary ESRD*: defines a TRA as:<br>• An admission with a principal* discharge diagnosis of CKD or ESRD<br>• A secondary diagnosis of anemia<br>• A procedure code for a RBC transfusion on the first or 2nd day of admission.<br>• A secondary diagnosis of anemia was not required when the principal discharge diagnosis was *International* Classification of Diseases (ICD-9: 285.21 - anemia in CKD).<br>2. *CBA 2 – Any ESRD:* defines a TRA as:<br>• Any discharge diagnosis for CKD or ESRD (primary or secondary)<br>• Any discharge diagnosis for anemia<br>• A procedure code for a RBC transfusion on the first or 2nd day of admission.<br>3. *CBA 3 – Primary Anemia*: defines a TRA as:<br>• An admission with a principal* discharge diagnosis of anemia<br>• A procedure code for a RBC transfusion on the first or 2nd day of admission.<br>4. *CBA 4 – Any Anemia*: defines a TRA as:<br>• A hospital admission with any discharge diagnosis for anemia<br>• A procedure code for a RBC transfusion on the first or 2nd day of admission.

TRA = Transfusion Related Admission , CBA = Claims Based Algorithm, CKD = Chronic Kidney Disease, ESRD = End Stage Renal Disease, RBC= Red Blood Cell

* The principal diagnosis is that which is determined to be responsible for the greatest length of inpatient stay (within the VHA).

Discussion: CBA Selection
Each of the four CBAs were designed to account for TRA coding variations seen in claims data. Despite CBA 1, representing the accepted coding procedure for a TRA, we anticipated CBA 2 would perform best at identifying both TRA and TRA-primary. This is because of the 'any' statement, in regards to both anemia diagnosis and ESRD/CKD diagnosis. A broader definition should ideally detect more cases than the more restrictive definitions which require either anemia or CKD/ESRD (CBA 1) as a primary diagnosis (CBA 3). Similarly, CBA 4 is inherently flawed outside of our pre-provisioned data because it does not require a CKD/ESRD diagnosis and would likely select patients that do not have CKD/ESRD.

Each of the four CBAs assume that CMS does not contain patient laboratory values. However, this assumption was based on legacy protocols that were modified in 2008. As of January 1, 2008, CMS required reporting the most recent hemoglobin/hematocrit levels with one of three modifiers (EA: ESA, anemia, chemo-induced, EB: ESA, anemia, radio-induced, EC: ESA, anemia, non-chemo/radio) for all Erythropoiesis Stimulating Agents (ESAs) claims.[6]While this change in protocol bolstered the amount of clinical data available, it only accounted for patients who received an ESA. This protocol was subsequently changed in the 2011 CMS Quality Incentive Program (QIP) which required all ESRD facilities to include hemoglobin/hematocrit values in their claims submission on or after January 12, 2012, regardless of ESA use. While more laboratory data was collected by CMS post-2011 this was not always the case during the course of our study period.[7]

We believed that inclusion of the CMS laboratory data would have created an incomplete picture given the difference in reporting policies across our study period, so we chose to code anemia only with available ICD9 codes (Table 14). Also, as mentioned by Wang et. al, CMS receives only one hemoglobin value each month for each patient's hemodialysis, which may not represent the patient's true hemoglobin status for the entire month.[8] However, given the availability of CMS laboratory data, the procedures described in the *CA* can be adjusted to run on more recent claims data. If this is done, the limitations described in Section 3.1 above should be considered.

Section 4: Sampling

Rationale: We found that TRA and TRA-primary accounted for only a small portion of our study population. This low prevalence necessitated sampling strategies that specially account for *imbalanced data.* This problem typically occurs when there is an extremely unequal distribution between classes of interest in the data, and one class is vastly underrepresented in comparison to the rest. When normal sampling schemes are applied to such data, it is highly unlikely that the underrepresented group will be selected unless much larger sample is used. Extremely low prevalence of a class of interest also affects the reliability of estimates like accuracy, in which case a substitute value known as the geometric mean is a viable substitute.[9,10] To account for this we devised a sampling strategy that supported population-level estimates of algorithm performance and at the same time provided insight into where errors may be occurring with the *CA*.

Sampling Strategy:
To define the different classes of interest used for sampling, we used variations of the *CA*, aimed at identifying a TRA and areas of potential error in our *CA*. This created four classes used in sampling the first was the basic definition of the *CA* as described in Section 2, used to define a TRA, along with three variations on the same concept. With a target accuracy statistically indistinguishable from 90% at the alpha=.05 level, we needed to sample at least 150 hospitalizations from sampling rule 1 for the lower end of the 95% confidence interval to be at least 85%. To achieve this required value as well as account for *imbalanced data*, we randomly sampled without replacement 400 hospitalizations from sampling rule 1, 200 from sampling rule 2, 200 from sampling rule 3 and 100 from sampling rule 4. The sample from rule 4 was small as we did not expect errors with this rule. Each rule is described in detail below. The 900 was randomly divided into four batches of 225 for review. To allow reviewer comparison, 10 charts from each reviewer were also reviewed by the other, for a total overlap of 20 charts. We used the first batch of 225 chart-review hospitalizations for error analysis and refinement of the *CA* and the exclusion criteria to adjust our algorithms as described below. After adjustment, 533 unique hospital admissions remained were used for the validation study and reported results.

1. *Sampling rule 1:* Is the primary class of interest and representative of the potential occurrence of a TRA in the data. This sampled class is the *CA* described in Section 2. It required evidence of anemia (HGB < 9.0 mg/dL or HCT < 27 %) (Section 1D) 24 hours prior to admission AND a RBC transfusion (section 1E) within the first 24 hours after admission. Reviewers determined the presence or absence of a TRA and TRA-primary during validation. During validation, if reviewers determined that the patient did not have ESRD-related anemia with a RBC transfusion within the defined time frames, or they showed evidence of exclusion criteria (Section 1C), this signaled the potential need to adjust our algorithms. This sampling class is representative of a true positive if correctly identified.

2. *Sampling rule 2:* Created to identify errors with our RBC transfusion definitions (Section 1E), it required evidence of anemia 24 hours prior to admission, with no evidence of a RBC transfusion within the first 24 hours after admission. During validation, if reviewers identified the occurrence of a transfusion in this class this signified the potential need to adjust our algorithms. This sampling class is representative of a true negative if correctly identified, as it is not a TRA or TRA-primary.

3. *Sampling rule 3:* Created to identify errors with our anemia data definitions (Section 1D), it required no evidence of anemia (HGB ≥ 9mg/dL or HCT ≥ 27%) between 24 hours prior to admission and 24 hours after admission, AND evidence of a RBC transfusion within the first 24 hours after admission. During validation, if reviewers identified the occurrence of anemia (HGB < 9.0 mg/dL or HCT < 27 %) in this class this signified the potential need to adjust our algorithms. This sampling class is representative of a true negative if correctly identified, as it is not a TRA or TRA-primary.

4. *Sampling rule 4:* Created to ensure we were correctly identifying patients who did not have any of the criteria of a TRA. This differs from the previous two values which are aimed at identifying partial TRA concepts to help identify sources of improvement in the algorithms. It required no evidence of anemia on admission AND no evidence of a

RBC transfusion during the first 24 hours of the admission. During validation, if reviewers identified the occurrence of a transfusion or anemia in this class this signified the potential need to adjust our algorithms. This sampling class is representative of a true negative if correctly identified, as it is not a TRA or TRA-primary.

Section 5: Statistical Estimate of Population-Level Parameters

Rationale: We intended to statistically evaluate the performance of each CA and CBA, with respect to the detection of TRAs. To do this, we had to make special considerations due to the sampling mechanism and the rare prevalence of RBC transfusions. The sampling mechanism described in Section 4 was implemented due to the rarity of RBC transfusions in the population, and resulted in four samples that, when combined incorrectly, would produce biased statistical estimates for the population (with the singular exception of positive predictive value (PPV), as screening positive was only possible for sampling rule 1). To obtain unbiased estimates for the population, we implemented inverse probability weighting (IPW) based on the probability of sampling from each sampling rule. As mentioned previously, the geometric mean (g) of sensitivity (SE) and specificity (SP) was considered an appropriate metric because the outcome is rare[9], and we wished to evaluate the effectiveness of the CA and CBAs based on their properties, rather than properties which are highly influenced by prevalence, such as accuracy.

Methods

Statistics calculated:

Using chart review results, we calculated several statistics for each algorithm: SE, SP, g, accuracy, PPV, negative predictive value (NPV), and incidence of TRA. These statistics were calculated using the TRA and TRA-primary RBC transfusion definitions. Sample-level statistics as well as population-level statistics were calculated, but sample-level statistics have not been reported because of the known bias.

Confidence intervals (CI) were calculated for each statistic. To obtain population-level estimates, all statistics were bootstrapped. Bootstrapping results in an approximately normal empirical distribution for each statistic, with an easily calculated mean and standard deviation, but calculating a 95% CI using this normal assumption for boundary values with high variance resulted in estimates that violated the boundaries on the statistics calculated (e.g. some were > 100%). To account for this, we performed a logit transformation to estimate all confidence intervals.[11]

Note about incidence: Incidence of TRA is uniform across tests because it reflects the weighted incidence for each of the four sampling rules, based on chart review results for TRA and TRA-primary. This is independent of each test.

Estimating Statistics using bootstrapped IPW:

N_i: The total number of hospitalizations in sample rule i;
n_i: The number of hospitalizations sampled from rule i;
P_i: n_i/N_i, The probability of sampling each hospitalization in sample rule i;
W_i: $1/P_i$, The weight for sample i;
TP_i: The number of true positives among n_i;
FP_i: The number of false positives among n_i;

TN_i: The number of true negatives among n_i;
FN_i: The number of false positives among n_i;

$$Population\ Level\ accuracy = \frac{\sum_{i=1}^{I=4} W_i * TP_i + \sum_{i=1}^{I=4} W_i * TN_i}{\sum_{i=1}^{I=4} W_i * FP_i + \sum_{i=1}^{I=4} W_i * TP_i + \sum_{i=1}^{I=4} W_i * FN_i + \sum_{i=1}^{I=4} W_i * TN_i}$$

$$Population\ Level\ PPV = \frac{\sum_{i=1}^{I=4} W_i * TP_i}{\sum_{i=1}^{I=4} W_i * FP_i + \sum_{i=1}^{I=4} W_i * TP_i}$$

$$Population\ Level\ NPV = \frac{\sum_{i=1}^{I=4} W_i * TN_i}{\sum_{i=1}^{I=4} W_i * FN_i + \sum_{i=1}^{I=4} W_i * TN_i}$$

$$Population\ Level\ SE = SE_{pop} = \frac{\sum_{i=1}^{I=4} W_i * TP_i}{\sum_{i=1}^{I=4} W_i * FN_i + \sum_{i=1}^{I=4} W_i * TP_i}$$

$$Population\ Level\ SP = SP_{pop} = \frac{\sum_{i=1}^{I=4} W_i * TN_i}{\sum_{i=1}^{I=4} W_i * FP_i + \sum_{i=1}^{I=4} W_i * TN_i}$$

$$Population\ level\ g = \sqrt{SE_{pop} * SP_{pop}}$$

Steps for Bootstrap Inference:
Step 1: For each sample rule i, generate a bootstrapped sample with size n_i by sampling with replacement in the original sample set.
Step 2: Calculate the above statistics using the bootstrapped sample, and store these results.
Step 3: Repeat steps 1-2 approximately 2000 times, then use the bootstrapped distribution for each statistic to calculate the mean and standard deviation.
Step 4: Apply a logit transformation to limit to [0, 1] when estimating 95% confidence intervals.[11]

Section 6: Document Abbreviation Legend

	Abbreviation	Definition
1	CA	Clinical Algorithm
2	CAPRI	Compensation and Pension Records Interchange
3	CBA1	Claims Based Algorithm 1
4	CBA2	Claims Based Algorithm 2
5	CBA3	Claims Based Algorithm 3
6	CBA4	Claims Based Algorithm 4
7	CDW	Corporate Data Warehouse
8	CKD	Chronic Kidney Disease
9	CMS	Centers for Medicare and Medicaid Services
10	CPRS	Computerized Patient Record System
11	CPT	Current Procedural Terminology
12	DSS	Decision Support System

13	ECS	Event Capture System
14	ESA	Erythropoiesis-Stimulating Agents
15	ESRD	End Stage Renal Disease
16	HCPCS	Healthcare Common Procedure Coding System
17	HCT	Hematocrit
18	HCUP CCS	Healthcare Cost and Utilization Project Clinical Classifications Software
19	HGB	Hemoglobin
20	ICD	International Classification of Diseases
21	IV	Intravenous
22	LAB	Laboratory
23	LBB	Laboratory Blood Bank
24	MCA	Managerial Cost Accounting
25	MCAO	Managerial Cost Accounting Office
26	RBC	Red Blood Cell
27	TRA	Transfusion Related Admission
28	TRA-Primary	Transfusion Related Admission -Primary
29	VA	Veterans Affairs
30	VBECS	VistA Blood Establishment Computer Software
31	VHA	Veterans Health Administration
32	VistA	Veterans Information Systems and Technology Architecture

Section 7: Appendix A References

1. Managerial Cost Accounting (MCA) [Internet]. U.S. Department of Veterans Affairs; [cited 2016 Dec 9]. Available from: https://www.herc.research.va.gov/include/page.asp?id=managerial-cost-accounting

2. Research Guide to the Managerial Cost Accounting National Cost Extracts [Internet]. U.S. Department of Veterans Affairs; [cited 2017 Apr 14]. Available from: https://www.herc.research.va.gov/include/page.asp?id=guidebook-mca-nde

3. Decision Support System (DSS) DSS FY17 User Guide [Internet]. U.S. Department of Veterans Affairs; [cited 2017 Apr 14]. Available from: http://www.va.gov/vdl/documents/Financial_Admin/Decision_Supp_Sys_(DSS)/dss_fy17_userguide_v1_1.pdf

4. MCA Other Data [Internet]. [cited 2017 Apr 14]. Available from: https://www.herc.research.va.gov/include/page.asp?id=other

5. VistA Blood Establishment Computer Software (VBECS) Version 2.2.0 [Internet]. U.S Department of Veterans Affairs; 2016 Nov. Available from: https://www.va.gov/VDL/documents/Clinical/VistA_Blood_Establishment_Com

puter_Software/vbecs_2_2_0_user_guide.pdf

6. CMS Manual System Pub 100-04 Medicare Claims Processing Transmittal 1412 [Internet]. Centers for Medicare & Medicaid Services (CMS); 2008 Jan. Available from: https://www.cms.gov/Regulations-and-Guidance/Guidance/Transmittals/downloads/R1412CP.pdf

7. CMS Manual System Pub 100-04 Medicare Claims Processing Transmittal 2311 [Internet]. Centers for Medicare & Medicaid Services (CMS); 2011 Sep. Available from: https://www.cms.gov/Regulations-and-Guidance/Guidance/Transmittals/downloads/R2311CP.pdf

8. Wang C, Kane R, Levenson M, Kelman J, Wernecke M, Lee J-Y, et al. Association Between Changes in CMS Reimbursement Policy and Drug Labels for Erythrocyte-Stimulating Agents With Outcomes for Older Patients Undergoing Hemodialysis Covered by Fee-for-Service Medicare. JAMA Internal Medicine. 2016 Dec 1;176(12):1818.

9. Kubat M, Matwin S. Addressing the curse of imbalanced training sets: one-sided selection. ICML. 1997.

10. He H, Garcia EA. Learning from Imbalanced Data. IEEE Trans Knowl Data Eng. 2009;21(9):1263–84.

11. Paoli B, Haggard L, Shah G. Confidence intervals in public health. Office of Public Health Assessment [Internet]. 2010. Available from: http://health.utah.gov/opha/IBIShelp/ConfInts.pdf
