# Supplementary material for: Validation of Algorithms Used to Identify Red Blood Cell Transfusion Related Admissions in Veteran Patients with End Stage Renal Disease
Source: EGEMS (Wash DC). 2019 Jul 3;7(1):23. doi: 10.5334/egems.257 (PMC6611485; doi:10.5334/egems.257)
Supplement: Appendix B. — Error analysis performed on algorithms prior to study implementation. [file egems-7-1-257-s2.pdf]

Appendix B. Batch 1 & Error Analysis

Summary

An error analysis was performed on the first batch of 225 chart-reviewed hospital admissions in order to identify classification errors and improve the performance of our clinical algorithm (CA). The charts used in the error analysis were not included in the validation study since we required admissions notes in the validation study to be independent of notes that initiated review and revision. To perform the error analysis, we identified false positives and negatives, investigated the cause of each of these errors, and implemented changes to address the errors discovered. The sampling rules were specifically designed to reveal deficiencies in the measurement of anemia via hemoglobin and detection of RBC transfusion; however, we found the primary source of error was the poor administrative coding of exclusion criteria, e.g., bleeding events due to gastrointestinal (GI) hemorrhage. Adjustment of exclusion criteria based on our error analysis reduced the number of hospitalizations available for the validation study but improved the measurement of exclusion criteria and the performance of the algorithms. The reduction in the number of available chart-reviewed hospitalizations diminished after implementation of new exclusion criteria but enough samples from each rule were retained to meet requirements specified in our power calculations.

Methods

Chart-reviews were divided into a “training” set (TS) which we reviewed to identify potential errors in the coding algorithms or exclusion criteria, and a “validation” set (VS) which was not used to enhance the algorithm or exclusion criteria. We maintained the independent VS to avoid *overfitting* our algorithms to the data – meaning we did not want our changes to reflect the charts sampled and not generalize to the full population.

During error analysis the data were analyzed in the following four steps:

1. Contingency tables were created, and performance of the clinical algorithm was evaluated with sensitivity, specificity, and measures of accuracy.
2. Cases of false positives or false negatives were identified, and subjected to clinician review.
    a. Chart reviewer comments were reviewed.
    b. Codes associated with the incorrectly classified hospitalizations were reviewed.
3. Changes to the study or CA criteria were investigated as potential solutions.
4. Changes in exclusion criteria and algorithm measures were made, and the TS data were reanalyzed.

The changes based on the TS analysis were retained if they were beneficial in identifying TRA/TRA-primary. The new criteria were applied to the exclusion criteria and clinical algorithm used in the final validation set of 533 chart-reviewed hospital admissions.

Results

The hospitalizations were divided such that the TS contained 225 hospitalizations, and the validation set contained 675. The TS contingency table and related statistics (un-weighted) are presented below. Note that our preferred metric for algorithm performance is the geometric mean, the square root of the product of sensitivity and specificity, which each contain information about CA performance.

1. Contingency tables

The contingency tables depict the ability of the CA to detect a TRA, as indicated by the clinician chart review. We see the TS sample has a geometric mean of 86.2%. Note that the 95% confidence intervals for statistics presented are calculated using the binomial exact method for all statistics except geometric mean, which is based on a logit transformation of the bootstrapped statistic.

Table 1: Contingency table of TS. Note 33 False Positives, and 4 False Negative

Test	Clinician: TRA	Clinician: Non-TRA	Total
CA: TRA	67	33	100
CA: Non-TRA	4	121	125
Total	71	154	225

TS, Training Set, TRA, Transfusion Related Admission, CA, Clinical Algorithm

Table 2: Statistics associated with Table 1

	Estimate (%)	95%CI LB(%)	95%CI UB(%)
Accuracy	83.56	78.157	87.83
Sensitivity	94.37	86.390	97.79
Specificity	78.57	71.437	84.31
PPV	67.00	57.305	75.44
NPV	96.80	92.061	98.75
Geometric mean	86.2	81.8	89.7

LB, Lower Bound, UB, Upper Bound, PPV, Positive Predictive Value, NPV, Negative Predictive Value

2. Review of false positives and false negatives

There were 33 cases of false positives and four cases of false negatives. These errors were investigated through clinician review of both the reviewer comments, and codes associated with each hospitalization. We found that of the four false negative cases, two were due to hemoglobin levels meeting anemia criteria that were not found by the sampling rules, and two were due to a procedure code for transfusion not having been recorded. Of the 33 false positives, most were due to the presence of blood loss related to other conditions, while nine were due to transfusions which were detected by the clinical algorithm, but which were not present in the blood bank records. Of these nine transfusions that were not present in blood bank records, five were detected only via evidence of RBC transfusion orders, while the remaining four were recorded as procedures. Attempts were made to correct for these sources of error.

Table 3: Sources of error discovered via in-depth review of the 37 cases with disagreement between the clinical rule and the chart reviewer

Count	Source of Error
2	False Negative: HGB < 9.0
2	False Negative: Transfusion not coded

1	False Positive: Bleed/injury
2	False Positive: Epistaxis
10	False Positive: GI bleed
7	False Positive: Hematological
1	False Positive: Hemoptysis
1	False Positive: Hemothorax
9	False Positive: No transfusion occurred
2	False Positive: Surgery

HGB, Hemoglobin, GI, Gastrointestinal

Note that study criteria had already excluded multiple ICD9 diagnosis codes for GI bleeding from the study. Our review of administrative codes revealed that GI bleeds often went un-recorded in discharge diagnosis codes, meaning there was evidence of GI bleeds in the medical notes but not affiliated ICD codes to document this condition.

3. Investigation of solutions: Revised Exclusion Criteria

The exclusion criteria were modified in an attempt to account for the false positives seen above (Table 3). Because discharge diagnoses failed to capture GI bleeding (which were excluded using ICD9 code), multiple timeframes to identify the associated ICD9 exclusion criteria were considered, and ultimately the window from 3-weeks prior to hospitalization, until discharge was selected after input from clinical experts. In addition to a longer GI Bleed timeframe, we also added more GI bleed ICD9 diagnosis codes, excluded patients receiving IV PPI during hospitalization, and patients with positive guaiac test during hospitalization (see Appendix A for details). To address other false positive caused by bleeding, we excluded admissions with records of surgical procedures.

These changes resulted in a reduction in the number of hospitalizations available for review but slightly improved the performance of the algorithm. The size of TS decreased from 225 to 188, but the Geometric mean increased from 86.2% to 87.1%. Despite this modest increase, we decided to implement these changes to identification criteria because it improved the completeness of the study exclusion criteria.

Also investigated was the impact of removing RBC transfusion orders as evidence of a RBC transfusion code on false positives. We found that removing orders decreased the performance of the CA by introducing false negatives and resulted in a reduction of the geometric mean from 87.1% to 86.6%. Consequently, this was not implemented as a change to the CA.

CARRIE STOPPED HERE

4. Revised contingency tables

After the implementation of the revised exclusion criteria above, the contingency table below was generated. Note that as mentioned above, bleeding often went un-coded, and consequently correction of the CA and study exclusion criteria was not possible. Of the 37 hospitalizations incorrectly classified, nine were eliminated from the resulting TS as the result of this work, and none were reclassified.

Table 4: Contingency table of TS post adjustment. Note 24 False Positives, and 4 False Negative

Test	Clinician: TRA	Clinician: Non-TRA	Total
CA: TRA	59	24	83

CA: Non-TRA	4	101	105
Total	63	125	188

TRA, Transfusion Related Admission, CA, Clinical Algorithm

Table 5: Statistics associated with Table 4

	Estimate (%)	95%CI LB (%)	95%CI UB (%)
Accuracy	85.11	79.32	89.49
Sensitivity	93.65	84.78	97.50
Specificity	80.80	73.02	86.74
PPV	71.08	60.57	79.73
NPV	96.19	90.61	98.51
Geometric mean	87.1	82.1	90.9

LB, Lower Bound, UB, Upper Bound, PPV, Positive Predictive Value, NPV, Negative Predictive Value

Discussion

This batch error analysis improved the study criteria, but incomplete coding practices prevented us from dramatically improving the CA performance. Regardless, we consider this approach to have improved the study criteria by enhancing detection of bleeding events and surgeries that represent alternative explanations for transfusions and subsequently excluding these hospitalizations from the study. Of the 225 TS hospitalizations, 37 were removed due to these changes. Of the 675 admissions reviewed for the validation study, 142 were removed due to these changes to study criteria. We consider this error analysis to have strengthened the cohort by eliminating hospitalizations with alternative explanations for RBC transfusion.
