# Supplementary material for: Validation of Algorithms Used to Identify Red Blood Cell Transfusion Related Admissions in Veteran Patients with End Stage Renal Disease
Source: EGEMS (Wash DC). 2019 Jul 3;7(1):23. doi: 10.5334/egems.257 (PMC6611485; doi:10.5334/egems.257)
Supplement: Appendix C. — Chart review procedures. [file egems-7-1-257-s3.pdf]

Appendix C. Chart Review Process: Establishing the Reference Standard

Purpose: Appendix C details the process by which our study implemented chart abstraction.

Outline:

Section 1. Chart Review Preparation: Form Evolution
A. Pre-Planning Chart Review
Section 2. Training Chart Reviewers
A. 'Clinical Expert: Reference Standard'
Section 3. Study Abstraction: 'Chart Reviewer Reference Standard'
A. Finalized Form
a. Table 1: Conditional logic justification and inclusion into the chart review form.
B. Date Ranges
C. Chart Review: Pre-Populated Data
a. Figure 1: Chart-review abstraction template used to create the clinical reference standard and perform data quality inspections on identification of RBC transfusions and laboratory values
Section 4. Chart Review Adjudication
a. Table 2: Final chart review adjudication results detailing the number reviewed by the clinical expert

Section 1. Chart Review Preparation: Form Evolution

Process: The form was revised and tested during a series of meetings with participation and feedback from clinical experts and chart reviewers. These meetings drove the chart review form interface and questionnaire evolution used to record relevant clinical measures during chart review process. After the preliminary chart review form was generated, beta testing was done as a team using 15 patient records. The chart review template went through 14 document iterations prior to the finalized form (Figure 2).

A. Pre-planning Chart Review

Rationale: The clinical and programming teams participated in meetings to define the clinical and data concepts needed to design and implement the chart review.

Clinical Expert Backgrounds
- Hematology/Oncology, MD
- Nephrology, MD
- Pharmacy, PharmD

Chart Reviewer Backgrounds
- Clinical Pharmacy, PharmD 15+ years
- General Surgery, MD 3rd Year Resident

Data Expert Backgrounds:
- Research Scientist: Epidemiology, PhD
- Data Scientist, MStat
- Computer Scientist/Software Developer, MS

Chart Review Form (Figure 2):

- **Purpose**: To guide reviewers though the chart abstraction and to efficiently capture responses in a standardized way.
- **Software**: Microsoft Access
- **Data Sources**: The chart review form was pre-populated with data identified based on sampling from 4 clinical rules. Data was obtained from a combination of the VHA CDW, CMS, and MCA (detailed in Appendix A).

Section 2. Chart Reviewer Training

Rationale: We provided reviewers with training and testing of TRA concepts to ensure they fully understood the goals of abstraction. Training also allowed the reviewers to become acclimated to the electronic chart review form and to receive technical as well as conceptual help from clinical experts and software designers. This process generated two reference standards that were used in our study, the 'clinical expert reference standard' and the 'chart reviewer reference standard'. The 'clinical expert reference standard' was designed to train and test chart reviewers, as described in Section 2A below, and the 'chart reviewer reference standard', which was established after training, was used in study analysis to determine algorithm performance, as described in Section 3 below.

A. 'Clinical Expert: Reference Standard'

Rationale: The 'clinical expert reference standard' was established, to allow us to test reviewers understanding of the abstraction parameters. This consisted of 53 records isolated from the population to establish a training set (3 records) and a testing set (50 records). The initial 3 records reviewed by experts were presented during a training session which was designed to outline the chart review process. After training, reviewers progressed to the testing phase where they independently reviewed the first testing batch of 25 patient records for comparison to the 'clinical expert reference standard'. Each reviewer was required to achieve an accuracy of 90% in order to begin abstraction for use in the study. If reviewers did not achieve 90% accuracy they were required to be retrained and retested on the 2nd testing batch of 25 records. However, both reviewers passed the first round. Reviewer performance was measured exclusively on TRA classification (question 3a/3b).

Training Session Topics presented by clinical experts included:

- Presentation of a TRA
- Presentation of a Non-TRA
- Chart Review Form Navigation
- CAPRI Navigation
- Info buttons

Section 3. Study Abstraction: 'Chart Reviewer Reference Standard'

Rationale: Study abstraction was completed in order to generate an algorithm comparator. The 'chart review reference standard' was used to determine the performance of one clinical algorithm and four claims based algorithms, as detailed in *Appendix A.* After training, reviewers progressed to chart review for use in the study. During this phase, 900 hospitalizations were reviewed. Each reviewer was assigned two batches of 225 admissions. To allow reviewer comparison, 10 charts from each reviewer were also reviewed by the other, for a total overlap of 20 charts. Of the four batches, one batch was used to refine and train the clinical algorithm, and the remaining three batches formed the 'chart reviewer

reference standard' used in this study. Chart-reviewers were required to use the national CAPRI system to view patient charts.

A. Finalized Electronic Chart Review Form

Rationale: The finalized chart review form was developed and used to capture the study's reference standard. This form was comprised of two types of questions, those that were designed to determine if data capture problems were occurring and those that defined the patient record as a TRA/Non-TRA. Conditional logic was included to require the minimum number of reviewer responses.

Conditional Logic: was included in the chart review form to streamline the process. This was modeled as a questionnaire hierarchy where preceding answers could determine the presence or absence of subsequent questions.

Table 1: Conditional logic justification and inclusion into the chart review form**

**Conditional Logic**	**Reason**
*Verification of the lowest hemoglobin (Hgb) value, question 1, was always asked. If the lowest Hgb value supplied was not correct reviewers were asked to record the lowest value listed in the patients record within the provided date range *(Question 1)*	If anemia did not occur neither could a TRA. However, the response to this question did not determine the presence or absence of any of the subsequent questions, in the event that the pre-populated value was not correct. Alternate Hgb values recorded by reviewers were assessed for date/time variations and data capture problems during analysis. Since this question was asked in Boolean fashion (yes/no), backend fixes were applied to records that did not have a hemoglobin <9 within 24hours of admission. This question did not preclude question regarding the presence or absence of a TRA/TRA-primary (Question 3a, 3b), because it did not require the reviewer to assess the exact time the laboratory value was recorded. Hgb values recorded by reviewers that were outside the specified time window or above the anemia cutoff were designated as non-TRA during analysis.
*Determination of RBC transfusion administration, Question 2a, was always asked after Question 1. If reviewers answer indicated there was no RBC transfusion administered, reviewers did not see questions regarding the cause of anemia or admission. ***If*** *question 2a = 'No Transfusion Administered during the 3 day period'* ***Then*** *No(3a, 3b)*	If a RBC transfusion did not occur the record did not fit the criteria of a TRA. Non-TRA was assumed.
If answer indicated a RBC transfusion was administered during one of the dates provided, reviewers did not see the question requiring them to identify the transfusion order date. ***If*** *question 2a ≠ 'No Transfusion Administered during the 3 day period'* ***Then*** *(3a) and No(2b)*	RBC transfusion administration was a primary component of a TRA. Orders were used as an indicator of administration. Requiring reviewers to record an RBC transfusion order, in the absence of an administration record, was deemed only necessary for use in error analysis to identify orders that were inappropriate or potential indicators.

If answer indicated ESRD related anemia was not the primary cause of the RBC transfusion, reviewers did not see the inquiry regarding the reason for admission. However, they were asked to record the cause of anemia from a dropdown box selection which included GI Bleed, Surgery, Hematological Disorders, Chemotherapy, None, Other-Please Explain. If ESRD related anemia was the primary cause of the RBC transfusion, reviewers had to determine if the anemia was the primary cause of the admission.<br><br>***If*** *question 3a = 'No'*<br>**Then** *No(3b) and 'Select the most likely cause of anemia'*<br><br>***If*** *question 3a = 'Yes'*<br>**Then** *3b*	This logic was developed to create a reference standard for two alternate TRA clinical concepts and to identify potentially missed exclusion criteria. These two definitions were termed *TRA* and *TRA-Primary*.<br>• *TRA* represented the broad concept in which patients with ESRD-related anemia were admitted for any reason (except exclusion criteria) and received a RBC transfusion. *(Identified by 3a= 'Yes')*<br>• *TRA-Primary* creates a rigid definition. These patients were admitted exclusively for ESRD-related anemia and subsequently received a RBC transfusion. *(Identified by 3b= 'Yes')*

*Reviewers had to answer Questions 1 and 2a as part of each patient record. This allowed us to determine if we were correctly identifying hemoglobin lab values and RBC transfusions.
**Below Figure 2 provides a visual of the chart review template

B. Date Ranges

Dates provided on the left of the chart review form (figure 2) labeled "Admission Date", "Start Date", "End Date" were used to help streamline abstraction by providing a relevant timeframe to filter the medical records in CAPRI.

Question (1) "Evidence of Anemia"
Timeframe:

- Admission Date
- Start Date: 1 day before admission date
- End Date: 1 day after admission date
  *The timeframe was used to identify the lowest hgb/hct level occurring during this window.

Question (2) "RBC Transfusion Administered" or "RBC Transfusion Ordered"
Timeframe:

- Admission Date
- 1 day after Admission Date
- 2 days after Admission
- No transfusion
  *This timeframe was used to account for potential differences that may occur between the data which contained a recorded time stamp, and the EHR record, which was represented as a date without a time stamp. Dates were built into the questionnaire to assist the reviewer with identifying relevant dates of interest which were relevant to the study criteria.

Question (3) "Evidence of ESRD Anemia Administered"
Timeframe:

- Admission Date
- Start Date: Admission Date
- End Date: 3days after admission date
  *This timeframe was supplied by the chart review form to help reviewers filter through a large number of medical notes. It was used exclusively to assist the review process. If the given date range was not sufficient to identify relevant notes, reviewers were allowed to extend the timeframe until they found notes which allowed them to sufficiently answer the question in relation to the admission date listed.

C. **Chart Review: Pre-Populated Data**
Rationale: Data used to pre-populate chart review forms relied on sampling methods centered around four predefined sampling rules. Random sampling occurred based on each sampling rule to ensure that potential records of a true TRA (*Sampling Rule 1*) was included in review along with potential areas of algorithm deficiency. The sampling strategy and sampling rules are discussed in detail in, *Appendix A section 4: Sampling*, however a limited definition is provided below.

Sampling Rules
Sampling Rule *1:* (TRA) Laboratory anemia with RBC transfusion within 24 hours of admission.
Sampling Rule *2*: (Non-TRA) Laboratory anemia with no evidence of RBC transfusion within 24 hours of admission.
Sampling Rule *3:* (Non-TRA) No laboratory anemia, but evidence of RBC transfusion within 24 hours of admission.
Sampling Rule *4:* (Non-TRA) No laboratory anemia, and no evidence of RBC transfusion within 24 hours of admission.

Figure 2: Chart-review abstraction template used to create the clinical reference standard and perform data quality inspections on identification of RBC transfusions and laboratory values.

Main

TRA Project Data Collector

Ver 0.4 - Feb 4, 2016

Sauer Team

The goal of this chart review is to confirm End Stage Renal Disease (ESRD) admissions have evidence of ESRD associated anemia prior to a RBC transfusion.

Patient SSN [redacted]

Admidatetime [redacted]

Facility [redacted]

Clinical Rule 1

How to change Facility and Patient in CAPRI

Evidence of Anemia

Admission Date [redacted]

Date range:

Start Date: [redacted]

End Date: [redacted]

Calculator

Question 1

Is [redacted] the lowest hemoglobin value between the start and end date provided on the left?

- (•) Yes
- ( ) No

How to locate Hgb labs in CAPRI

RBC Transfusion Administered

Question 2a

Please choose the date of the first RBC transfusion ADMINISTERED during the provided time frame:

- ( ) [redacted] (Admission Date)
- ( ) [redacted] (Admission Date + 1 day)
- ( ) [redacted] (Admission Date + 2 day)
- (•) No Transfusion Administered during the 3 day period

How to locate RBC transfusions in CAPRI

Reference: First Transfusion Date

RBC Transfusion Request

Question 2b

Please choose the date of the first RBC transfusion ORDERED during the provided time frame:

- ( ) [redacted] (Admission Date)
- ( ) [redacted] (Admission Date + 1 day)
- ( ) [redacted] (Admission Date + 2 day)
- (•) No Transfusion Ordered during the 3 day period

How to locate RBC transfusion Order/request in CAPRI

Evidence of ESRD Anemia Admission

Admission Date [redacted]

Date range:

Start Date: [redacted]

End Date: [redacted]

Question 3a

Is the anemia for which the transfusion was given mainly related to ESRD?
Causes not associated with ESRD would include GI Bleed, Bleed due to injury, Surgery, Chemotherapy, hematological disorders, etc.

- YES
- NO

How to locate Discharge Summary, H&P, Nephrology Note

Evidence of ESRD Anemia Admission

Admission Date [redacted]

Date range:

Start Date: [redacted]

End Date: [redacted]

Question 3b

Was the anemia for which the transfusion was given the primary reason for admission?
Please view the discharge summary for any alternative causes for admission.

- YES
- NO

How to locate Discharge Summary, H&P, Nephrology Note

Optional Comments

Prev Next Save

First Created On:

Latest Modify On:

Created by:

Modified by:

Section 4. Chart Review Adjudication

Rationale: Adjudication was performed as part of the chart review process, in order to ensure adherence to the TRA study classification. Due to the broad nature of the TRA definition, as seen in the distribution of principal diagnosis codes (Manuscript Table 6); clinical experts had concerns regarding reviewer's adherence to classification of a TRA. The study defined TRA occurred when anemia, with a RBC transfusion, was primarily caused by ESRD, in the absence of exclusion criteria (e.g. GI bleed, surgery, hematological disorders, chemotherapy, or bleeding due to injury). If the stated exclusion criteria were not present in the medical note then the reviewer should have determined that the primary cause of the anemia was ESRD. As an example of this decision making process; there were records where the reviewer commented as having anemia due to multifactorial causes, this was still considered a TRA as long as those causes did not include exclusion criteria.

Adjudication Process: Records were selected for adjudication if the reviewer indicated that ESRD was not the primary cause of anemia and left a comment detailing why they made this determination. The hematology/oncology clinical expert then evaluated these records along with the reviewer comments to determine if the reviewer classification should be maintained. Adjudicated results were used in final analysis. This process only considered the classification of TRA and not TRA-primary.
